# Part II. Spirit of the Foreign Periodicals

**Published:** 1833-07-01

**Authors:** 


### Spirit of the Foreign Periodicals.

We purpose henceforward to devote an allotted and considerable space to this department, in order that our readers may see at a glance what their Continental neighbours are about. It is not that every fact thus narrated is important, nor every opinion true; but when information is so generally diffused as it is now—when we exercise such an influence upon the Continent, and the Continent reciprocally on us, it is surely a matter of laudable curiosity, if nothing more, to learn their sentiments on common things, and view the varieties of practice that varieties in education, habits, and institutions generate. It is more convenient for our readers, though less so for ourselves, that these scraps of foreign literature should be brought together and assembled in one place; for thus, at one coup d'œil, they may be said to command a prospect of a vast extent of country, instead of having it broken into numerous fragments by intervening obstacles. It is true that there may be less variety, but there is more consistency, and, in our opinion, a wider and a more extended utility. Without further preface, we shall pass at once to the matter before us.

I. Selections from a Report of the Labours of the Medical School of Abouzabel. Published by M. Clot Bey. Marseilles, 1832.

The majority of the diseases prevalent in Egypt originate in, or are connected in some measure with, irritations of the gastro-intestinal canal, and hence the therapœia of the Broussaian or physiological school is admirably fitted for their cure. Before my arrival, it was little known; but now the most sceptical have been converted to its truth; and the two great endemics of the country, viz. dysentery and ophthalmia, have especially illustrated its superior efficacy. Even the plague, at least the milder forms of it, has been brought under its sway; for although this pestilence does not always prevail epidemically, I am satisfied that it appears every year under the form of an intense gastro-enteritis, which is followed by buboes, or, as the Arabians term them, "hiars."

Scabies, tinea, and syphilis are common; but gonorrhœa is rare. Lepra, elephantiasis, hepatitis, mania, ague and rheumatism, and palpitations of the heart are among the diseases most frequently seen. The small-pox is almost always imported by the negroes from the interior of Africa.

Pleurisy, pneumonia, and phthisis are rare; and I have often remarked that strangers, who arrive with pectoral complaints, are always much benefited, and often cured, during their sojourn. M. Clot suggests the propriety of European physicians sending their patients to Egypt, instead of to Italy, Switzerland, &c. Rickets, aneurism, and cancer are infrequent. Among the surgical cases requiring operations are enumerated 58 of lithotomy, of which number only 6 were fatal; 20 of amputation, including one at the hip-joint, which, however, was unsuccessful; 4 of strangulated hernia; 1 of the removal of an enormous tumour, weighing 110 pounds, of the scrotum, and another of a carcinomatous tumour of the lower jaw, a great portion of which required to be cut out—both of these were fortunate; 2 cases of trepanning of the sternum, from which the patients recovered perfectly. Diseases of the eye are exceedingly prevalent, and especially the different forms and complications of cataract, pterygion, glaucoma, and staphyloma. Cataract is, in by far the greater number of cases, caused by preceding ophthalmia: the Arabians have, from time immemorial, practised an operation which resembles somewhat that of couching. Hydrocele is a very common disease; M. Clot states that upwards of 200 cases were operated upon in the hospital of Abouzabel during the space of seven years, and calculus of the bladder is far from being infrequent. The great success of my operations, says M. Clot, I attribute to the favourable climate of the country, to the temperament of the Arabians, which is little irritable, and also to their inaptitude for moral suffering. I have never seen tetanos follow any wound or injury, although my readers may probably know that it often did so in the French army, during their stay in Egypt.

It is worthy of record, that the homoiopathic mania has reached even the shores of the Nile. One of the apostles of this strange creed offered to the Viceroy, that he would instruct 30 of the pupils of our school for a reward of 30,000 talaries. At first he obtained a share of credit, and was permitted to make a few trials in the military hospital of Cairo. He selected nine cases of ophthalmia, taking care that in all of them the active stage had passed away; and also several cases of subacute dysentery, which had already been treated by antiphlogistic remedies; but, in spite of these very favourable circumstances, little or no benefit was afforded to the patients; and very soon the homoiopathic doctor was obliged to try his fortune elsewhere.

We are informed that, for many years after the foundation of the medical college of Abouzabel, there was no pharmaceutical school there, in consequence of a government one having been established at Cairo; but it has been subsequently deemed more prudent to transfer to Abouzabel both it and also the veterinary school, which had been founded at Rosetta, in the year 1243 of the Mahometan era. One good effect of these changes is, that the students have it now in their power to attend to natural philosophy, botany, chemistry, pharmacy, comparative anatomy, and veterinary medicine, during their stay at Abouzabel. In consequence of the austerity of Moslem manners, which do not allow males to assist or be present at accouchements, M. Clot contemplated the institution of a class for the instruction of midwives; he presented a report of his sentiments to the Pacha, and Mahomet-Ali forthwith ordered 10 female slaves to be educated for the purpose. M. Clot anticipates much good from this very recent establishment, and says, "We shall no longer see the most simple cases in midwifery followed by death, nor hear that presentations of the leg or of the arm require the child to be mutilated to permit its birth. A "maternité" and a foundling hospital are also to be established immediately. Besides these very important changes, our indefatigable author has projected a plan of a magnificent college or university, to be erected in Alexandria, which, from its natural and acquired advantages, is so much superior to Abouzabel, and where the earliest and most celebrated school of antiquity flourished—that school which had for its pupils Aristotle, Herophilus, Erasistratus, and Galen.

Appended to the memoir, is the report of a proposition which the medical Bey has communicated to Mahomet-Ali, on the subject of the re-population of Egypt. In the time of the Greeks and Romans, the number of inhabitants was from 10 to 12 millions—it is now reduced to three millions. This great falling off has been owing to frequent wars and revolutions; also to the prevalence of the plague, slavery, celibacy, polygamy, and pæderasty, and to the large military force, which has been kept up constantly of late years. One million of inhabitants cannot, in justice, support permanently more than ten thousand soldiers; but the Viceroy has had so many wars to wage in Haggiar, Sennaar, the Morea, Candia, and Syria, that he has been obliged to retain an immense standing militia; and we must remember that the population of the country suffers not only from the numbers slain, but also by the rest being kept away from their homes. The plan which M. Clot suggests, is to import a vast number of negroes from the interior of Africa, or from Cordofan, Sennaar, Darfour, &c. and that these negroes should be employed as domestics and in-door servants, in the place of the natives, who should hence be encouraged rather to devote themselves to agricultural pursuits. This importation would be a vast burden to the State, if defrayed by it alone; but M. Clot proposes that all persons in easy circumstances should be obliged to purchase their own servants, and that these servants must be always taken from the black population. Government might also enlist them in the army, and in this particular imitate the example of the Emperor of Morocco, who has a militia of a hundred thousand Negroes alone. Every Negro should be obliged to marry at the age of 25, in order that they might contribute to augment the population!! Our author is aware that he may be scandalized as an advocate of slavery; he abjures such a reproach, and founds his defence on the fact that these very Negroes whom he proposes should be imported into Egypt, are slaves already in Sennaar and Darfour, where they are badly treated and cruelly used. Moreover, says he, it is generally admitted that the Turks are by no means harsh masters, but rather that they treat their servants as they do their children, and almost always give them their liberty in course of time. As a matter of course, such as were enlisted would be treated as the other soldiers; and to the credit of the Pacha, several Negroes have already attained the rank of lieutenants and even of captains in the army.—*Trans. Medical.*

---

### II. Professor Roux' opinion on the Cause of Death after very Painful Injuries.

A man was admitted into the La Charité Hospital, with an immense tumour on the upper and inner side of the thigh. The operation for its removal was very protracted and painful, in consequence of the deep adhesions to the ossa pubis and ischii. The patient died on the third day, in a state of alternate stupor and delirium. On dissection, the only morbid appearance observed was, an effusion of serum into the lateral ventricles of the brain. M. Roux stated, that in almost all cases where death is consequent upon very severe suffering, he has found an effusion of serum either between the membranes, especially between the arachnoid and pia mater, or in the lateral ventricles. He has very often noticed it in patients who have died from burns, and more frequently in children than in adults, who can better resist pain.—*Ibid.*

---

### III. Severe Scald of the Mouth and Fauces from Boiling Liquor Potassæ.

A man engaged in the manufactory accidentally sucked in a mouthful of the boiling caustic ley. The effects were most dreadful; but under the judicious treatment of M. Bouillaud, who ordered repeated local and general depletions, he completely recovered.—*Ibid.*

---

### IV. Amputation at the Hip-joint.

This most dreadful operation was performed by M. Blandin at the Hôpital Beaujon, in January, 1832. The patient was a young female, and the following is the report of the diseased state of the limb—we give the particulars to enable our readers to judge for themselves of the propriety of the operation. "The right knee was horribly hypertrophied, to four times at least its natural size; the swelling extended up the thigh as far as the trochanter, and down the leg, but became gradually less and less at a distance from the joint; the pain was dreadful, and defied all sedatives; the skin had become so thin by distention that it seemed ready to burst, and the superficial veins were much enlarged and very tortuous. The evening before her admission into the hospital, while turning in bed, the os femoris snapped at the lower third of its shaft, and this accident was followed by an aggravation of all the sufferings. M.M. Blandin and Marjolin concurred in the propriety of amputation at the hip. The femoral artery was first secured, a large internal flap made, and the divided arteries tied; the limb being then forcibly abducted and drawn down, the bone was removed from its socket, and the outer flap made by one sweep of the knife. Six sutures were used to retain the edges together. On the fifth day there were appearances of sloughing of the wound, and the discharge was unhealthy. Death took place on the tenth day.

*Examination of the Amputated Limb.* The femur was diseased in all its length; both trochanters were involved, and even the neck of the bone was softened, and bathed with a sanies, which had seemingly dissolved its spongy texture; the condyles had become enormously enlarged and were also much softened; but the shaft of the bone was dryer and more brittle than in health. The soft parts were so completely changed that it is almost impossible to render any intelligible description of them; "tout était pêle-mêle," and was bathed, or rather macerated in an abominable sanies.

*Dissection of the Body.* Partial reunion of the stump had taken place, but here and there it was sloughy; the flaps had adhered above and below the acetabulum, leaving this uncovered and forming a large cavity for suppuration; two large fistulous canals extended upwards along the glutei, and another still larger than either of these ran up within the sheath of the iliacus internus as high as the middle of the iliac fossa; at one part the great sciatic nerve, to the extent of an inch, was surrounded with pus. A purulent collection was found in the pelvis, and communicated with the surface of the stump; the internal iliac vein was inflamed, softened, and contained pus. The viscera of the abdomen were healthy; the lungs at some parts presented the appearance of tuberculous matter infiltrated through their texture. M. Blandin was of opinion that it was not truly tuberculous matter, but rather the effect of certain changes induced by the purulent secretion from the inflamed iliac vein admitted into the general circulation. What corroborated this view was, that the liver presented patches of diseased alterations almost identical with those observed in the lungs. Now M. Blandin has found that in cases of ordinary phlebitis the liver is, of all organs with the exception of the lungs, the viscus most frequently the seat of purulent deposits.—*Ibid.*

*Note.* Many of our readers will probably agree with us, in considering the operation in the preceding case as improper and unwarrantable; and for this very plain reason; the disease had extended as high, if not higher than the hip-joint; and the incisions must have been made therefore through diseased parts. The description of the state of the limb before the operation, and the examination of it afterwards, leave no doubt on our minds, that an English surgeon would not, in such a case, have put his patient to the agonies of the knife. We strongly deprecate the thirst for operations, so common among many of the Continental surgeons. ED.

---

### V. IGNORANCE OF A FRENCH HOSPITAL PHYSICIAN.

M. Pigeaux, in a memoir on blood-letting in diseases of the heart, observes —"almost constantly I have remarked that the treatment of Valsalva, when followed with all its rigour, has rendered the general condition of the patients worse; and more particularly in five cases which occurred at the La Charité, and of which I have kept notes; in these the severe depletions very evidently accelerated the fatal termination of a disease, which in two at least of the five, proved, on dissection, to have been only simple chlorosis!"—*Bullet. de Therap.*

---

### VI. VARIOLA AFTER VACCINATION.

Dr. Terzachi reports that, from Feb. to August, 1831, 748 cases of variola were admitted into the Milan Hospital; that of these, 614 had been previously vaccinated; and that 45 cases proved fatal, or 7½ in the 100. Dr. Tinella has lately published an account of an epidemic variola which prevailed at Mantua; he is quite satisfied of the beneficial effects of vaccination; but strongly urges the propriety of repeating the operation every tenth year. If the vaccine virus be introduced simultaneously with or immediately after the infection of variola, the vesicles of the one and the pustules of the other disease, are formed together, and pass through their different stages, unaffected by each other.—*Annali Univ.*

---

### VII. Use of Turpentine in Sciatic Neuralgias.

M. Martinet has adduced a long catalogue of cases to shew the superior efficacy of small doses of the oil of turpentine. To prevent its acrid effects on the stomach and bowels, he recommends that it be always blended with some corrective excipient, such as honey, gum-arabic, magnesia, yolk of egg, &c. and the dose ordered is a drachm or two in divided doses, daily. As a matter of course a correct diagnosis must have been previously made, in order that we may be satisfied that there is no inflammation or organic disease of the nerves; under such circumstances we cannot reasonably expect a cure from the turpentine alone. If the drug vomits or purges to excess, opium should be added. In 40 cases of acute neuralgia, 34 were cured, 5 relieved, and one was not benefited. In 31 chronic cases, 24 were cured, 3 were relieved, and 4 experienced no advantage: 33 of the cases treated with the turpentine had resisted other remedies previously employed. The period generally required for the cure was from 5 to 12 days; in a few cases the medicine must be continued longer. Out of 58 cases 48 were sciatic, 3 crural, 4 brachial, and 3 facial neuralgias.—*Bull. de Therap.*

---

### VIII. On the Re-production of the Crystalline Lens, after the Operations for Cataract.

We shall confine ourselves to describing shortly the actual observations and experiments narrated in the memoir of M. Mayer, and refer our readers for more extended details to the January number of the Archives Generales and Journal Complementaire, which have formed a new-year's marriage, and are to be associated in future. He examined the eye of an old woman, on whom the operation of couching had been performed several years previously. There was no trace of the depressed lens; the vitreous substance occupied its place, and immediately behind the anterior wall of the crystalline capsule, was observed the posterior wall or layer with the vitreous humor pressing forward upon it. The researches of others do not however agree with the statement, and M.M.Cottreau and Leroy d'Etioles have always found that the lens was really and perfectly reproduced in animals, after the operation of extraction. The following experiments, among many others, were performed by M. Mayer.

The lens was extracted from the left eye of a rabbit, which was killed three days afterwards. No trace of a new lens was found at this period, nor on the 4th, 5th, 6th, or 7th days; but on the 8th, the crystalline capsule contained a small ring of crystalline substance, which could be separated from the capsule. At the end of one month a large ring of crystalline substance occupied the place of the removed lens. In another rabbit, examined about the same time after the operation, a large white annular lens, with an opening in the centre, was found in the capsule, which adhered to this new lens. In eight weeks the new crystalline presented several white granular points arranged in a circle, having an opening in the middle; and in four months and a half it was not yet completely regenerated; for it was deficient at the centre, leaving there a rounded aperture, at the place where the capsule had been cut during the operation.

Soemmering has given us an account of four dissections, at different periods after the operation on the human subject.

In the first, the patient had been couched eight years and a half before his death. In the place of the crystalline capsule two semilunar whitish cheesy formations were formed, attached by their peripheral margin to the zonula Zinnii, and floating free at the inner margin; they were doubtless the remains of the crystalline capsule. The new crystalline was transparent, gelatinous, and imperfectly formed. The former one had been completely absorbed, but a small piece of the original capsule was found imbedded in the vitreous humor.

CASE 2.—*Three Months after Couching.* In the place of the former lens Soemmering observed an annular transparent gelatinous deposit, imperfect at the centre, which was occupied with a fine, almost diaphanous and arachnoid membrane, situated right behind the pupil, and forming a septum between the aqueous and vitreous humors.

CASE 3.—*Two Years after Couching.* Similar appearances were discovered. A ring of transparent substance, of the consistence of jelly, in the situation of the lens of the left eye; in the right one, which had been also operated on, the new deposit was only semicircular, the upper part of the circle being deficient. Probably the cause of this was that, during the operation, the upper half of the capsule had been completely torn from its adhesions.

CASE 4.—*Three Years after Couching.* The annular "renflement," or new deposit, had been very regularly formed; it was slightly and equally convex on both its surfaces, and was quite free from any adhesions to the uvea.

---

It is to be kept in mind that in order to display the annular crystalline substance the eye must be immersed in strong alcohol, by which the new deposit is rendered slightly opaque. Soemmering was at first puzzled to determine whether it was really a substitute for the removed lens, or was merely a product of inflammation; but he was speedily satisfied that the former was the case. Sometimes the ring is imperfectly formed; and in other cases we find only isolated points or grains. These cannot be the debris of the original cataractous lens, as some have imagined, for the simple reason that these grains are perfectly transparent, and the cataract was opaque. The preceding facts sufficiently shew that there is a reproduction, although an imperfect one, of the crystalline lens; but we have reason to believe that an indispensable condition is a sound and healthy state of the capsule, and especially of its front layer; if this be either much torn and destroyed, or if it be rendered opaque by disease, there is no regeneration of the crystalline. In all probability, the secretion of the new substance is chiefly, if not altogether, from the inner surface of the anterior wall or layer of the capsule; and as this layer adheres intimately to the contained crystalline, no traces of the cavity or liquor of Morgagni can be henceforth discovered. The process of regeneration proceeds invariably from the circumference to the centre; and is always found interrupted at the place where the capsule has been cut, or lacerated during the operation;—the rent in the capsule is occupied with cellular substance. Hence the crystalline substance is never entirely reproduced, but always presents in the centre, or opposite to the injured part of its capsule, an opening which is filled up with a fine cellular tissue. The shape of the new crystalline is generally that of a three-quarter moon, the horns of which nearly touch each other. In the experiment on the rabbit, which was allowed to live for four months and a half after the operation of extraction, the new crystalline had this form, with a free space in the middle, occupied by a cellular web.

M. Leroy d'Etiolle and Soemmering state that they have found the new crystalline free and unadherent to its capsule; the observations which I have made do not coincide in this respect with theirs;—it is a point left open for examination. It is worthy of remark, that the mass of the new crystalline almost always exceeds that of the original; but that the entire eye very generally becomes somewhat shrunk and contracted for some time after the operation. This shrinking is found to extend even to the optic nerve, and that, too, beyond the decussation as far as the thalamus. It is conjectured however, that in favorable cases the eye and its appendages may resume their original volume.—*Journ. Complem.*

### IX. Lisfranc's Treatment of Amaurosis.

First of all, we should ascertain whether there are any symptoms of inflammatory fulness and activity in the eye or head;—as a matter of course, such cases require depletion; when, however, we have reason to believe that the disease is one rather of debility, Lisfranc strongly advises us to direct our attention in an especial manner, to stimulate the frontal and other branches of the fifth pair of nerves by means of repeated blistering over the eyebrows and temples. Should this fail, we must endeavour to excite the torpid organ by acting immediately on the ciliary nerves, any irritation of which is speedily propagated to the ophthalmic ganglion and the origin of the trigeminus. This is most effectually done by the application of stimulants to the cornea; and of these stimulants the nitrate of silver in substance is the best. The inferior segment of the cornea is to be lightly touched, till we perceive a whitish cloud;—the eye is then to be immediately washed with water. Considerable pain is felt; the whole apparatus of the eye is put into a state of so increased activity, that on the morrow a stranger might suppose that our patient laboured under acute ophthalmia. This treatment induces sometimes vomiting; and as it always occasions temporary contraction of the pupil, it must not be employed when there is a tendency to this evil. The operation requires to be repeated several times.—*Ibid.*

### X. Obliteration of the Uterine Veins after Puerperal Fever.

M. Duploy has communicated an interesting paper on the pathology of metro-peritonitis or puerperal fever, and has adduced several observations to prove that the veins of the uterus become plugged up, and finally reduced to ligamentous cords, in cases of puerperal fever which have terminated favorably. As the attention of our readers was drawn in our last number to the morbid anatomy of this interesting disease, [vide review of Dr. Lee's work,] it is most useful to collate and compare the researches of the continental physicians with those made at home. Unfortunately, says M. Duploy, most of our patients have died from the effects of the absorption of the pus secreted from the uterine veins, with all the symptoms of complete typhus. On dissection, the vessels have been found lined with false membranes, and gorged with a purulent fluid, either alone, or mixed with blood. But where the patients have survived for a considerable time after the symptoms of the fever had been subdued, we have found the uterine veins in a very different condition. On making an incision through the substance of the uterus, we have observed several yellowish grey lines or bands traversing it in various directions; these bands proved on minute dissection to be obliterated veins—they have been noticed chiefly towards the sides or margins of the uterus. In all the cases where these appearances were found, the patients had previously laboured under symptoms of metro-peritonitis which had been subdued. We are therefore warranted in concluding that uterine phlebitis is not necessarily and invariably fatal, but is occasionally, as well as phlebitis occurring elsewhere, cured by the obstruction and subsequent conversion of the inflamed veins into impervious cords. Probably this fortunate issue is only to be expected when the affected vessels are small and few in number. I have never found the trunk of the hypogastric, or of the ovarian vein, completely obstructed. The following cases illustrate the above remarks.

*Case* 1. A woman, aged 19, was delivered at the Maternité on the 1st March, 1829. Symptoms of puerperal fever set in next day and continued for a week, but were at length subdued; an obstinate diarrhœa however wore out her strength, and she died on the 10th of April, forty days after her confinement.

On dissection, the uterus was found to be well contracted upon itself, of a pale fleshy colour, except in the lateral parts of its anterior surface, which were of a deep brown, and marbled as it were with patches of ecchymosis.—When cut into at these points, numerous veins were found full of blood; but at the margins of the uterus, and just in front of the attachment of the broad ligaments, we observed small greyish cylinders or bands, which ramified through the substance of the organ; and on accurately tracing these, we were satisfied that they were obliterated veins.

*Case* 2. A servant, aged 17, was delivered on the 5th of February, and died on the 16th of the same month, with all the symptoms of malignant puerperal fever. On dissection the uterus appeared at first sight to be tolerably normal; but on cutting through its substance, near the margins, or lateral edges, the vessels were discovered to be full of solid concreted pus, somewhat resembling a false membrane, which quite obstructed their canals.

*Case* 3. A woman died three weeks after delivery. The uterus was found to be adhering anteriorly to the omentum, and posteriorly to the rectum; its inner surface was black, as if ecchymosed; its tissue not softened; when cut into, numerous grey bands were observed, passing in different directions; these bands were the veins which had become plugged with a firm tenacious substance.

The preceding three cases illustrate the author's positions;—the following is a striking specimen of a very intense uterine phlebitis extending even to the vena cava.

*Case* 4. A woman, aged 22, was delivered at the Maternité on the 14th April, 1829. The symptoms of puerperal fever were ushered in with violent shiverings on the following day. Active depletory measures were employed, but without avail; and she died comatose on the 27th.

*Dissection.* Well-marked ramollissement in the right hemisphere of the brain;—the uterus exteriorly appeared normal; its inner surface however was lined with a coating of concreted pus, resembling a pseudo-membranous deposit. On the left side, some of the uterine veins were filled with pus, their coats hardened; on the right side, all the uterine veins and roots of the ovarian veins presented the same appearances; the trunk of the ovarian vein, along its whole length, to its embouchure into the cava, contained purulent matter; its walls were much thickened, and almost of a fibrous consistence, and lined with a false membrane; the vena cava was greatly distended, and plugged up with a large clot, which at its upper and lower extremities was merely sanguineous, but at the middle, corresponding to the entrance of the ovarian vein, presented a dirty grey, half-bloody, half-purulent appearance.

---

### XI. Observations on the Morbid Anatomy of other Viscera in Puerperal Fever.

LYMPHATIC SYSTEM. Many suppose that it requires very minute dissection to discover the morbid condition of the lymphatics of the womb;—it is quite a mistake. When they contain pus, they are seen immediately under the peritoneal coat, like greyish white lines, alternately distended and contracted, on the anterior and posterior surfaces, and on the lateral margins of the womb; sometimes even along the broad ligaments to the ovarian veins, round which they form a complete network; occasionally, they are so much dilated at points, that they might contain moderate-sized peas. In a few cases, they may be easily traced as far as the lumbar glands, which, when cut into, are then found softened and soaked with pus. M. Tonnellé twice found purulent matter in the thoracic duct. The suppuration of the lymphatics exists sometimes alone, sometimes simultaneously with a similar state of the veins. In all the cases in which we have found, on dissection, the lymphatics in a diseased state, the symptoms during life were those of typhus, but not of such an aggravated type as accompanies the suppuration of the veins, in consequence, probably, of the communication with the circulating mass of blood being not so immediate in the former, as in the latter case.

The Fallopian tubes present generally appearances of active inflammation; they are found swollen, œdematous, invested with lymph, and adhering to the surrounding parts; when cut open, they sometimes contain purulent matter, which may be squeezed along their tubes, through their fringed extremities, into the cavity of the peritoneum, and thus occasions (so says Cruveilhier), peritonitis. The ovaries are variously affected; they become red, and, as it were, ecchymosed; sometimes they are atrophied, but more frequently much enlarged in bulk; this increase may be owing to a serous infiltration, to a suppuration of their substance, or to a general deposition of lymph; sometimes nothing remains but their investing membrane, and the ovary forms one entire abscess; at other times, there are numerous smaller ones between its lobules; the pus may be either fluid, or so nearly concrete as to crumble between the fingers. Both ovaries are in most cases affected, although in different degrees. In five cases only, out of 105 dissections, were the lungs found diseased; their substance presented the red and grey hepatization, and in one example numerous vomicæ were discovered, such as are often observed in the bodies of those who have died of ordinary phlebitis. In a great number of cases, I have found the mucous coat of the stomach quite pulpy and decayed at particular points —in one case a perforation existed. M. Desormeaux has repeatedly met with spontaneous perforations of the stomach, in women dying in childbed. M. Dugès has observed them in 10 cases out of 266 patients, who died of puerperal fever at the Maternité. He regards it as an effect rather of chemical softening and solution, than of ulceration or gangrene; and formed his opinion on the absence of any marked gastric symptom during life, and of any adhesions or redness around the wound —on the softening and thinning of such parts of the small intestines as are bathed with the brown mucous matters effused round the perforation; and on the mode in which the destruction seems to be propagated by simple contiguity, and not at all by the continuity of structure. I have thus observed, says he, in a case where a perforation existed near to the large end of the stomach, the adjacent portions of the liver and spleen softened, brown, and deprived of their serous coat; the diaphragm, and even the pleura, perforated opposite to the opening in the stomach; the mediastinum partially dissolved, the lungs softened and laid bare, where they had been in contact with the brown viscid sanies. These spontaneous perforations may, therefore, be regarded as the effects of a true chemical solution of the parietes of the stomach. The blood in the heart and large veins is generally found thin and pale, and the crassamentum is loose and easily broken down.—*Journ. Complem.*

---

### XII. On Hydatids, and their Conversion into Tubercles.

M. Kuhn has lately read before the French Academy a memoir on acephalocysts, and the manner in which these parasitical productions give rise to tubercles. He holds the opinion of Laennec, Bremser, and others, that they are to be considered as truly of an animal nature; and draws a distinction between those found in the human body, from what are often seen in sheep and other lower animals; the former, says he, are always propagated by internal buds, or growths which are thrown off from the inner surface of the original hydatid, and may be, therefore, denominated "endogenous;" they may be compared to a nest of boxes, one within the other, whereas the latter produce buds only on their outer surface, and are, therefore, "exogenous." It was after a very careful examination of the lungs of oxen, which had died of a species of phthisis called "pommelière," that M. Kuhn was led to the belief of the degeneration or conversion of hydatids into tubercles. The hydatids, by their irritation, cause cysts to be formed around them; these cysts become stronger, fibrous, or even cartilaginous; meanwhile, the acephalocysts enlarge by serous imbibition, and multiply by buds from their inner surface; these again, in course of time, give rise to others, the whole nest being contained in one bag. From the inside of this bag is secreted a yellowish viscid matter, which becomes thicker and thicker; M. Kuhn regards it as the primitive tuberculous deposit: it gradually solidifies, and, with a simultaneous shrinking of the cyst, tends to squeeze and kill the enclosed animals, thus giving rise to a nucleus of tubercles. Sometimes the tubercles are not entirely filled up, but are hollow, and we observe only the shell or dried husk of the acephalocyst; we may even separate the thin layer of the animal from the debris within, by immersing some of the tubercles in water. M. Kuhn has enriched his memoir with beautiful illustrative drawings; they throw much light on the etiology of the tubercles which are found in the lungs and liver of ruminating animals. The co-existence of hydatids and tubercles, in the same organs, is a fact at once curious and most interesting. The subject is one of much importance, and deserves future examination.—*Révue Medicale.*

---

### XIII. New Speculum Uteri, and Cure of some Cases of Sterility.

M. Melier has lately suggested an improvement on the speculum uteri; it consists in adding a solid moveable cylinder of smooth wood, enclosed within the speculum, and projecting with a rounded head beyond its vaginal extremity. The great advantage of this is, that the folds of the vagina are readily extended without any pain, and the instrument is easily conveyed up to the cervix uteri; the inner cylinder is then withdrawn. M. Melier strongly insists upon the good effects of injections, conveyed into the cavity of the womb, in many diseases; and as barrenness is, no doubt, very often the consequence of a neglected inflammation of the cervix uteri, he suggests the propriety of using them "pour rendre féconde des femmes jusqu'ici steriles." In one case, M. Melier, by means of his improved speculum, detected an engorgement of the cervix uteri; and, as he considered that this was the only obstacle to conception, he advised local emollients, discutients, and leeching, soon after the use of which the lady became pregnant of her first child.—*Journ. Hebdom.*

---

### XIV. Induction of Premature Labour by means of a Sponge and by Puncture.

*Case* 1. Is that of a woman, aged 31, in whom the pelvis was too small to permit the passage of a full-grown child. Premature labour was induced in the eighth month. A plug of sponge an inch long, and of the size of a goose's quill was cautiously introduced into the cervix uteri, and left there. In the course of three hours, some short pains were felt; they alternately returned and disappeared till the evening, when the plug was withdrawn by means of a thread secured to it. The operation was repeated four times, and on the third evening labour regularly commenced; both mother and child did well.

*Case* 2. The patient, a young female, aged 17, was the victim of very severe "eclampsia puerperarum," which threatened a fatal termination. Professor Lovati decided on bringing on premature labour; he punctured the membranes with a trocar, and discharged the liquor amnii. The labour did not commence till 29 hours after the operation, and the patient ultimately recovered.

*Remarks.* The professor prefers generally the employment of the sponge, as in the first case, to puncturing the membranes, because the escape of the water, before labour comes on, frequently renders that tedious and painful. He objects to the methods of frictions over the uterus, of irritating the cervix uteri, and of separating the bag of membranes from its attachment round the cervix. He, however, admits that in cases which require very prompt delivery, as in the second one reported, we must resort to puncturing the membranes, as the use of the sponge is too tedious.

It is rather singular that the operation of inducing premature labour is inhibited by law in France.—*Annali Univ.*

### XV. Malignant Carbuncle in Italy.

Dr. Gullo informs us that, in Calabria and other provinces of Naples, the cattle-dealers, butchers and others of the lower classes are frequently afflicted with a very dangerous carbuncle, known by the name of the "carbuncolo tristo." It appears at first as a round pustule, not unlike that of vaccination, of a livid red colour, black in the centre, the edges elevated and covered with vesicles. It often proves fatal in three or four days, if neglected. The cause is the direct introduction of a putrid virus into any pricks or wounds when cutting up the cattle for market.

Dr. G. has found the following treatment most successful:—he makes several incisions on the edges, and a deep one through the centre of the carbuncle, and after wiping away the blood and sanies, sprinkles the wounds freely with powdered corrosive sublimate; lays on a plaster composed of four grains of the same mixed with the yolk of an egg. In 24 hours a deep eschar is formed, suppuration begins, and in seven or eight days the ulcer is healed.—*Ibid.*

### XVI. Extemporaneous Vesication.

M. Pigeaux recommends the following method. Apply a dossil of lint, well wet with spirits of wine, to the part, and set fire to it. In a few seconds the epidermis will be found to be detached, and we can then remove it with our nail. The operation is very speedy and not painful.—*Revue Medical.*

### XVII. Remedies against Scrofula.

Hufeland very highly lauds the good effects of the Æthiop's mineral, or black sulphuret of mercury, combined with a little magnesia and tinct. rhubarb. Along with occasional baths it constitutes the treatment which he has found to be by far the most efficacious in strumous affections of the skin, enlarged glands, ophthalmias, and intestinal complaints.

Boyer, and other eminent French surgeons have much faith in the internal and extenal use of the subcarbonate of potass. The solution is employed as baths, lotions, and injections; when exhibited inwardly, it is advantageously combined with the tincture of gentian. —*Ibid.*

### XVIII. Insanity in Italy.

Mental derangement is much less common in Italy than in more northern countries, as the following table shews.

| | Population. | No. Insane. |
|---|---|---|
| Italy ........ | 16,789,000 | 3,441 |
| France ...... | 32,000,000 | 32,000 |
| England...... | 12,700,000 | 16,222 |
| Wales........ | 817,148 | 896 |
| Scotland...... | 2,093,454 | 3,652 |
| State of N. York | 1,617,458 | 2,240 |

We must admit that there is no doubt a great number of insane persons in different parts of Italy who are not in any asylums, and are therefore not included in the number of 3441, but even supposing that there is a third more to be added, the proportion is very strikingly inferior, and is a proof, among others, that insanity is the less prevalent in a country, as it is the more tranquil and the less agitated by the wants and desires of civilization. In Turkey, Egypt, Russia, &c. the number of lunatics is very small, and in France and England very large. It is greater in the North than in the South of Italy. Pellagra is a very common cause of it.—*Journ. Complem.*

---

### XIX. TWO EXTRAORDINARY CASES OF FASTING.

Dr. Schmalz, of Dresden, in a former No. of Hufeland's Journal, has related two very singular examples of abstinence from all food, protracted for an almost incredible length of time. We must remember, however, that he saw both individuals, and had an opportunity of personally ascertaining the particulars, and moreover the first case was the object of a Government enquiry.

Angelica Vlies was born in the neighbourhood of Delft, in South Holland, on 20th August, 1787. In her early years her constitution was very feeble and delicate, and she was much subject to cramps, induced by intestinal worms, which she voided both upwards and downwards in great quantities. She enjoyed tolerable health till 1811, about which time she was first seized with violent hysterical paroxysms; during these the bowels were obstinately confined. Subsequently she had repeated attacks of chronic enteritis, and her appetite, which had been throughout very sparing, now began to fail altogether. At one time better, and at another time worse, she continued in the above state till May, 1818, when she discontinued the use of solid food entirely, and took nothing but drinks, chiefly whey. All medicines were rejected by vomiting as soon as swallowed. For upwards of four years she tasted nothing solid, with the exception occasionally of a little fish and salad, which she sucked, but never swallowed. In the Spring of 1822, the attack of hysteria became so violent as to threaten death; an enema was given on the 10th of March; the bowels and also the bladder were then relieved; and this was the last time that any regular evacuation of stool or of urine took place. About this time she refused all nourishment whatsoever, fluid as well as solid; and now the catamenia which had hitherto been regular, although scanty, ceased. She frequently moistened her mouth with a little cold water to abate the burning heat she felt there. In July, 1822, an erysipelas appeared on the abdomen; it was relieved by the constant use of bread and milk poultices. In the following year she had a severe attack of dyspnœa, and fixed pain in the left side of the chest. Her physician, Dr. Grootenbeer, ordered a blister. In 1824 she had repeated seizures of subacute arteritis; in 1825 these seizures were neither so frequent nor so severe. In October of this year she voided, after most excruciating suffering, a small quantity of urine and fæces; during 1826 she made urine twice, and at each time only a few drops. Thus, from the 10th March, 1822, to this period she had had relief only once by stool, and three times by urine. The Dutch medical commission were very anxious at this time to induce her to remove to the Hague, in order that an opportunity might be had of strictly inquiring into her case; she would not however consent to this; but permitted four nurses to wait upon her alternately for the space of a month; the expense of their attendance was defrayed by Government. Soon afterwards a memoir was drawn up by Dr. Vorstman, and published at Delft, 1827. According to the authentic reports of the nurses, Angelica took no food, fluid or solid, from Nov. 11th to Dec. 9th. During this time, she used to moisten her mouth with water, tea, or whey; but she invariably spat the fluid out again, and the quantity was thus frequently somewhat increased, and certainly never diminished; she had no evacuation by stool or urine, but had occasionally belchings of wind. During the days, she sewed and amused herself with reading. She rose, or rather was lifted from bed, at 9, a.m. and was carried back at 11, p.m.; but she slept very little, being much distressed with headache, swoonings, and cramp. Her age at this time was 41, but her appearance indicated more than 60 years, her face being shrivelled, and her eyes dull and lustreless; her tongue was clean and dry, the skin was parched; the pulse normal in frequency, but exceedingly weak and small; the sensibility of the cutaneous, and perhaps also of the deeper nerves, was so much impaired, that she was scarcely aware of her skin being pinched or pricked. Every hour and a half she was seized with a shivering, followed by a convulsive, lateral agitation of the head; these fits lasted generally for about two minutes.

Dr. Schmalz (the reporter of the case) visited her in Sept. 1828, and had an opportunity of being perfectly satisfied with the truth of the preceding statements; she told him that she had not eaten nor drunk any thing since the report of the medical commission, nearly two years before; and if we go back, we shall find that this extraordinary abstinence had now lasted six years and a half, from March, 1822. The patient told Dr. S. that she would very willingly take food, if she could in any way swallow it, but that this effort was impracticable to her. Here the report ceases, and Angelica was still alive at the date of the report.

Case 2.—*History of a Female who lived upwards of 2½ Years without Food.* Professor Ricci, of Turin, has published a full detail of this case in the Repertorio di Medecina, di Chirurgia et di Chimica di Torino.

Anna Garbero, aged 40, had hitherto enjoyed moderately good health, although her appetite had been always remarkably sparing; her food consisted generally of vegetables only once a day, and the bowels were not usually relieved above twice a week. Gradually the appetite became less and less, and once she passed 40 days without touching any solid or fluid aliment. But it was not till Sept. 1825, that a total inappetence for food came on; it was after a very scanty meal, consisting of only a mouthful or two of cabbage and a draught of wine and water, that she was seized at once with intense gastralgia, which continued for some time, till copious vomiting was induced; from this date she was unable to swallow any thing, and even her spittle was thrown back when she tried to allow it to pass down. Up to the 7th of the succeeding January, she neither eat, drank, nor had any relief by urine or by stool: the only appreciable evacuation was that of the catamenia, which, though very sparing, returned regularly.

Dr. Schmalz visited her at this period; he found her so emaciated, that she seemed a mere skeleton, over which a dry skin had been forcibly stretched. The skin was almost quite insensible to pricking, or to the strongest pressure; the limbs were cold and corpse-like; the pulse small and scarcely perceptible, but yet regular in frequency. The patient was quite willing to make an effort, at any time desired, to swallow food, but it was of no avail; and at length the mere sight of any victuals, however simple, brought on most painful vomitings. Things continued so till the end of June, at which time she became insensible and lethargic; this state of apathy continued till the 25th of the following November, when she quite suddenly and unexpectedly recovered her senses and speech. Her strength became weaker and weaker, and finally was exhausted in death on the 19th May, 1828.

The body was examined in the presence of Professors Rolando and Gallo, by whom a very interesting memoir was published at Turin; we give only the more interesting and illustrative details. The omentum majus was found drawn strongly downwards, and had become adherent to the brim of the pelvis, thus leaving the small intestines quite uncovered. This change had been caused by the falling down of the transverse colon, which was lying in the pelvic cavity; it was distended with hard fæces; the small intestines were, on the contrary, contracted to mere cords. On carefully tracing the colon, it was found that the canal of the descending portion was so much obstructed by the swelling of its mucous lining, that the fæces could only with difficulty be forced along; the obstruction was still greater at the commencement of the rectum, and completely prevented the transit of any solid matters. The contents of the ascending colon were more fluid, of a dark green, meconium-like colour, and most intolerably fetid; two lumbrici and several ascarides were found in the bowels.

The rationale or etiology of the preceding case appears sufficiently simple. We conceive that a chronic inflammation of the colon and rectum had been originally caused by exposure to the inclemencies of the weather, for the patient was a beggar; thus, not only was the appetite directly impaired, but also the passage of the feculent matters obstructed, and the general health became more and more deranged in consequence; complete anorexia was the consequence of the accumulation of the fæces; the colon was dragged down by the weight, and, at the same time, the stomach and œsophagus were necessarily displaced in a similar direction, and this displacement must have seriously injured their functions. Besides, traces of a slow inflammation of the mucous coats of the small bowels, and also of the stomach, were found upon dissection; and our readers need not be reminded of the effects which we daily observe to flow from such a morbid state. In short, we are to regard the preceding case as one of the melancholy results of neglected sub-acute enteritis, originally of the rectum and sigmoid flexure, and subsequently of the rest of the canal.—*Journ. der Pract. Heilkunde.*

---

### XX. Case of Extraordinary Congenital Bulimia.

Anne Denise was born in 1786. From her earliest years her appetite was most voracious, requiring more than four times the allowance of other children. She menstruated at 7 years of age, and, at this period, all the other attributes of puberty were developed. As she grew older, her appetite became more insatiable; she was dismissed from school because she devoured the food of her schoolmates. She therefore gave lessons herself, and the only reward that she wanted for her instruction was meat and bread. At this time she eat 10 lbs. of bread daily. She could not, however, make sufficient by her present employment, and, therefore, she engaged as a servant in an hotel. Several times she had been arrested for stealing loaves from bakers' shops; and at length she was reduced to beggary, as no person would keep her in their employment. She used to wander about the streets in Paris, devouring all the refuse of food which she found at different doors. A great variety of remedies had been ineffectually employed to overcome this morbid hunger. She was admitted into the La Salpetrière under MM. Esquirol and Amussat, for relief from epileptic attacks, to which she had been for several years subject. At that time she consumed from eight to ten pounds of bread daily; she drank very little. The bowels were confined; and she had two or three attacks of hæmatemesis every month. Occasionally her appetite became prodigiously increased, and at these times she would devour 24 pounds of bread in the course of a night; she was literally mad with hunger at these periods, so that if she was thwarted, or food refused, she would begin to chew her clothes, or whatever she could get hold of. During these paroxysms of bulimia, the epigastrium was found to be tender, and this tenderness was increased by pressure, and a profuse vomiting of blood generally ensued. This "grande faim" recurred only once a year, and always on the 9th of February. In the course of 24 hours, she has been known to have devoured 32 pounds and upwards of food; eating and vomiting blood alternately, till she fell down quite exhausted. M. Rostan, in 1819, tried various means, chiefly antiphlogistic, with only temporary benefit: ice was administered inwardly, and, for a time, considerably abated the fury of her hunger.

In 1823 she consulted M. Descuret, (the reporter of the case,) with the view of ascertaining whether there were any intestinal worms; he administered purgatives;—several pieces of tænia were expelled. The appetite was considerably diminished, and she was satisfied with 5lbs. of bread, and two or three basons of soup daily; and the "grande faim" did not take place this year, and indeed did not return until 1828.

As her hunger decreased, she became intolerably addicted to the abuse of spirits, which in time brought on such a depravation of appetite, that she would devour the raw lights of the slaughtered animals, and afterwards literally brouse upon grass. In July, 1828, having gone to her "favourite pasturage"!! she collected a quantity of grass and buttercups (ranunculus acris), which she eat for supper. During the night, she was seized with torturing pains of the abdomen—jaundice ensued, and she died in a few days.

*Dissection.* The stomach was small; the mucous membrane of it, and also of the intestines, was inflamed in patches, but otherwise healthy; the liver was very large; the other viscera sound. The condyles of the inferior maxilla were literally worn away!! For further particulars, we refer our readers to the October No. of Broussais'* *Annales de la Med.*

---

XXI. Arteritis and Spontaneous Gangrene of the Right Lower Extremity—Arteries and Veins plugged up with Coagula.

A girl, aged 17, previously in good health, was suddenly seized with shiverings, severe pains in the right leg, and especially in the foot of that side; the pains were so severe, that the patient compared them to tearing the nails from the flesh: in a few days the temperature of the limb began to lower and the foot assumed a blueish hue; her sufferings were not at all abated, in spite of bleeding and repeated leechings, &c. She entered La Charité about a fortnight after the first seizure. The constitutional symptoms were those of general feverishness and malaise; and the pains in the foot, leg, and lower part of the thigh were so intense, that the slightest motion caused her to scream out;—the skin of the toes and instep presented some purplish blotches, and when felt by the hand, the temperature of the limb, up nearly as far as the knee, was much lower than that of the other one. No pulsations could be perceived in the anterior tibial artery on the instep, nor yet in the posterior, tibial, peroneal, and popliteal arteries; they were, however, sensible at the upper part of the thigh. Bleeding, general and local, emollients, and opiates were prescribed, but without relief; the blood when examined was of a gooseberry-jelly colour, and stained the linen with pale-red spots. The purple blotches extended up the limb, and the temperature became still lower. The constitutional symptoms soon assumed a more formidable aspect; the breathing was short and anxious; there was intolerable anguish and repeated vomitings and hiccup, and no sleep could be procured by any sedatives. Cramps and pains were felt also in the left limb, which was swollen and tender. On the fourth day after her admission into the hospital, the whole right foot was of a uniform brown colour, the epidermis was peeling off, and a gangrenous odour arose from it. She died on the following day.

*Dissection.* The right foot was of a port wine colour at some points, and at others was perfectly black, especially around the toes, where the skin was hard and dried like leather; the subcutaneous cellular tissue was infiltrated with serum as far up as the lower part of the thigh; the muscles of the foot and leg were quite soaked with it, and resembled much the appearance of half decayed flesh. The left limb was also œdematous. The blood-vessels on the right side presented the following appearances; the crural artery from the groin to the ham was converted into a hard cord, whitish outwardly, and lined and plugged up with a dirty white friable coagulum, which at some points adhered to the inner surface of the tube. Similar appearances were found in the rami perforantes of the femoral, in the two tibials and in the fibular arteries; the internal surface of all these vessels was of a marked livid red colour; the inner coat was not however lacerable,

---

* It is stated that the phrenological development of this woman's cranium, at the organ of alimentiveness, was unusually large.

nor very evidently diseased; the vasa vasorum were not more developed than natural, and the surrounding cellular tissue was healthy. All the veins of the right foot were plugged up with coagula, some of a deep black, others of a greyish colour; the lower third of the internal saphena was also obstructed similarly. On tracing up the external saphena, the tibial and fibular veins of the crural, and even along the femoral, and external, and common iliacs to their junction with the vena cava, softened coagula was found in all, partially filling up their tubes.

The large nerves of the right limb were much redder than usual, and seemed as if injected with venous blood. The arteries of the left limb were sound, but the veins from the foot up to the common iliac, and even to its junction with the vena cava at different parts of their course contained softened broken down coagula.

The medullary substance of the brain presented the curious appearance of circular red circumscribed patches at various parts; in each of these circles the central point was of a darker hue than the circumference, so that they were not unlike to petechiæ on the skin. The lungs were œdematous; the pulmonary veins contained fibrous clots, which adhered feebly to the walls of these vessels; the right ventricle of the heart was occupied by one large coagulum, which had all the appearance of gooseberry jelly. Numerous small petechial spots existed on the pleuræ, costalis et pulmonalis.

*Remarks*. We have observed that the lining surface of the arteries of the right limb was found reddened, but that there was no other sign or mark of morbid change in it. It has been much disputed by pathologists whether we are to admit this appearance as a test of preceding arteritis: Haller, Meckel, Bouillaud, Broussais, and others contend that it is; whereas, Corvisart, Laennec and Hodgson, Andral, &c. are of a different opinion, and assert that it is a "cadaveric phenomenon."

The following valuable observations are taken from the article "Arteritis," in the Dictionnaire de Médecine, et de Chirurgie pratique. "The redness may be wanting in true inflammation of the arterial tubes; and on the other hand it may be often observed, where no inflammation had ever existed; we not unfrequently see it in examining bodies which are partially putrid; and in these the imbibition of the bloody serum is no doubt the cause of the redness. Thus, we are not to consider the redness and swelling as pathognomonic morbid phenomena; nor yet, should they be wholly discarded. It must be admitted, however, that in by far the greater number of cases of arteritis, the redness, if it does exist, is not caused by the injection of the vasa vasorum, but rather by a tincture, or as it were a fixing of the colouring matter of the blood on the internal surface of the vessels; and that therefore this inflammatory blush does not essentially differ from the cadaveric imbibition."

Cruveilhier is of the same opinion as Bouillaud, the author of the article in question; he does not consider the mere presence of a red colouring of the inner coats of the arteries as characteristic of inflammation; we should find at the same time a pencilled injection of the vasa vasorum in the cellular coat of the vessels, and also coagula adhering more or less firmly to their inner surface; it is this last appearance which, according to Cruveilhier, is to be depended upon chiefly. Gendrin, Delpech, and Dubreuil, state, that in arteritis, the lining surface of the vessels is red, has lost its glistening smoothness and polish, is somewhat rough or wrinkled, and may be readily detached, and that the other tunics are swelled and softened. The subject is still open to difference of opinion. Cruveilhier, as stated above, considers that the essential or pathognomic character of inflamed arteries is, that the blood within them is coagnlated.

But we must be on our guard, lest we are led to believe that this change in the blood is found in all inflamed arteries; this is certainly not the case, as is fully established by M. Barde, in the first vol. of the Revue Medicale, and by M. Bouillaud, in his treatise on fevers. On the contrary, Haller expressly states — "In vasis etiam vivi corporis sanguis coit," and the truth of the remark is confirmed by every one.

M. Alibert, in his inaugural thesis, gives it as his opinion, that in cases of gangrene with arteritis, the formation of the clots precedes, and actually occasions by their irritation, the inflammatory state of the lining membrane of the arteries. Several very interesting examples are detailed in this thesis; in the 2d and 3rd cases softening of the brain was found, with the morbid changes in the veins of the mortified extremity; and in the latter of these two cases an adherent clot was found in the left auricle; in another case, a clot was found in the pulmonary artery, and in a subsequent one, these sanguineous concretions existed not only in the vessels of the sphacelated limb, but also in the aorta, and in all its branches given off below the diaphragm; and in the common, internal, and external iliacs. Besides the venous trunk on the surface of the brain, and of the dura mater contained coagula; and at the upper and back part of the right hemisphere, a large black spot, two inches at least across, was observed; the texture of the brain was here exceedingly softened, and quite of a creamy consistence.—*Archiv. Gener.*

---

### XXII. Quinine successfully used against Toothache.

Toothache is not unfrequently periodical, and will continue to harass a patient long, if the usual anti-odontalgics are only used; many a sound tooth has been extracted, without the least relief to the suffering. Quinine, in such cases as are obviously intermittent, will almost always cure the pain as rapidly, and as effectually, as it does an ague. In short, all diseases in which there are periodic invasions and remissions, even in hectic, and in intermittent fevers dependent upon urinary disorders, the bark seldom fails to relieve for the time, if it cannot effect a cure.

---

### XXIII. Preservation of Leeches by Feeding them with Sugar.

The attention of the Academy of Medicine has been lately called to this subject by a chemist. A commission was appointed to investigate particulars, and they have given in their report, which, however, is not favourable to the proposal.

The chemist was of opinion, that the blood which we so frequently find in the water in which the leeches are kept is not disgorged, but flows from the wounds which the animals inflict on each other when huddled together; the commission doubt the accuracy of this. A great error has very generally been committed, in supposing that one of the causes of the loss of so many leeches, is the putrefaction of the "mucosities which exude from their bodies;" now these, so called mucosities, are in fact the epidermes, which are regularly thrown off at intervals, in the same manner as the scarf-skin of a snake. The impressions of the rings of the leech are quite obvious on this mucosity; it is detached first towards the head, and the animal escapes from it as from a sheath, which still adheres for a short time to the tail, so that we often see the leeches swimming about with this membranous appendage.—*Bullet. Gen.*

---

### XXIV. Gangrene of the Lungs.

A female was admitted into La Charité under Professor Bouillaud, with symptoms of severe bronchitis. Active depletion arrested the disease, and she was speedily restored to health; but a second attack, within a month, obliged her to return to the hospital on the 24th of January last. On the following day, the extreme fetor of the sputa and of the breath of the patient, when she forcibly expired, induced M. Bouillaud to suspect gangrene of the lungs; the fetor was so horrible, that chloride of lime was required to be kept constantly round her bed. She was largely bled and leeched, but the sputa retained their offensive smell. On the 4th Feb. she expectorated an immense quantity of fetid sputa; there was then the greatest prostration of strength. On the 11th, the inside of the mouth was covered with aphthæ and diphtheritic crusts, and in the evening she vomited a large cupful of clotted blood. She died on the 13th.

*Dissection.* Adhesions of both pleuræ; left lung presenting the ordinary appearances of bronchitis; in the right lung between the middle and lower lobes, near to the spine, we found a large pouch, of the size of a hen's egg, filled with greenish-black, half fluid, half clotted matter, a mixture, apparently, of blood and of the detritus of the substance of the lungs—the gangrenous stench was quite intolerable. The walls of this cavity were of a brownish-black colour, and the surrounding tissue was hepatised, and all the bronchi in the neighbourhood were inflamed. The trachea, larynx, epiglottis, and part of the pharynx were covered with pseudo-membranous, or diptheritic laminæ, of a deep green and purple colour, and which were easily rubbed off.—*Journ. Heb.*

---

### XXV. SUDDEN DEATH FROM PARALYSIS OF THE LUNGS.

The German authors attribute to this cause many of those instances of rapidly-fatal dyspnœa, which not unfrequently occurs during the course of other diseases, especially of phthisis. It is not uncommon for a medical man to leave his patient moderately comfortable, and apparently free from any immediate danger; and yet, in the course of a very few hours after, to be summoned to witness his death from complete strangulation. Dr. Shaeffer, of Ratisbon, first employed the appellation of pulmonary palsy to denote this affection; Storck called it catarrhus suffocativus, and Kerksig asthma paraliticus. It is common among infants, but still more so with old people. M. Lobstein regards many of the cases reported by Andral (who was at a loss how to explain their fatality) as instances of this disease. The following is an example.

A young man, aged 28, was admitted into the Strasburg Hospital with symptoms of general fever. Bleeding was ordered, and performed at 9 o'clock in the morning. At this time, there was no marked distress in the breathing; two hours afterwards intense dyspnœa came on, and this was accompanied with a strong mucous râle; the dyspnœa was speedily aggravated to orthopnœa; a severe pain and inward heat were felt along the entire length of the spine. The bleeding was repeated, with some relief to the symptoms, but the patient died soon after, quite asphyxiated. On dissection, no satisfactory morbid appearances were found.

Two other similar cases are reported; they occurred in phthisical patients. M. Louis, in his great work, "Recherches Anatomico-pathologiques sur la Phthisie," enquires—"how shall we explain so sudden a death, when there has been no apparent accident, nor any precursory nor concomitant phenomenon?" We answer that it is not necessary to discover indurations, hepatization, engouement, or ulcerations, upon dissection, but that we must remember that the lungs are vital organs, and that their vitality may become suddenly affected by paralysis.—*Archiv. General.*

---

### XXVI. FATAL PURPURA HÆMORRHAGICA; PETECHIÆ IN THE BRAIN, HEART, LUNGS, &c.

This case occurred in a man aged 32. The constitutional symptoms were those of the most appalling prostration, and the hæmorrhages from the nose and bowels were most profuse, and could not be arrested.

On dissection, there were found numerous petechiæ dispersed over the medullary substance of the brain; each red point was surrounded with a circle of greyish matter. The pleuræ were spotted over with petechiæ; the lungs were generally œdematous, and here and there contained bloody deposits; the external surface of the heart presented numerous petechiæ; there was little blood in the cavities, and that was thin and pale. The parenchyma of the liver was soft and pale; and in the centre of its substance was found an extravasation of blood—a sort of " foyer apoplectique."—*Ibid.*

---

### XXVII. Double Vision with one Eye.

M. Prevost, the distinguished professor of Geneva, and Mr. Babbage, are annoyed with this irregularity of vision. Whenever they look at an object, without straining their eyes, they see two images, one situated above the other. In the case of the latter philosopher, the upper image is more faint and indistinct than the lower, or true one, and they are separated by an angle of 12 degrees. When his health is deranged, the upper or false image becomes more distinct, but the angle of separation is not changed. In consequence of the smallness of this angle, the image is not ordinarily seen quite double, but there is rather a confused border perceived round the real object; when he looks through any small aperture, the feeble image disappears, and the same effect is produced by inclining the head backwards, and directing the sight somewhat under the eyelid; also by contracting the eyebrows, or by using a concave lens.

The affected eye of M. Prevost sometimes sees three images of the same object, one above the other. He readily ascertained that the highest image corresponded with the most inferior picture on the retina, by moving slowly a screen before his eye, from above downwards; he thus observed that the inferior image disappeared before the other, and, as it faded, the upper one became more distinct. Sometimes he could hide the images by the eyelids—the upper image by the lower lid, and the lower image by the upper lid. M. Prevost was at first much annoyed by this double vision, for when he was reading, the " o" always seeming " 8," &c. By using a convex lens, he found that, by using it at a certain distance from the eye, only one image was seen, but it was surrounded with a light shade; when the glass was withdrawn further, the two images returned, and the same took place when it was brought very near to the eye; but, in the latter case, the images were seen side by side, and not the one above the other. M. Prevost is of opinion that, in such cases of double vision as his own and that of Mr. Babbage, the crystalline lens is chiefly at fault, so that it becomes a double refractor; this condition may be induced by a fracture, bruise, a partial flattening of its surface, or by a separation of one of its layers. The effect of a fracture is readily witnessed in a glass lens; for, if broken, we at once perceive two images. Dr. Wollaston attributed to this cause his own defect of vision, and found that the error was for the time corrected, by looking through the refracting angle of a prism steadily at the object. It is not necessary that the lens be actually broken; one of its segments or laminæ may be somewhat displaced and irregularly inclined, and thereby the double refraction may be caused. In the memoirs of Dr. Holyoke, who died at the age of 100 at New Jersey, in 1829, it is stated that, for several years, all objects were quadrupled or quintupled; when he looked, for example, at the moon, he saw five moons.—*Annal. de Chemie.*

---

### XXVIII. Medical Monomania.

When will physicians cease to localise every disease, and to insist upon charging a solitary ganglion, a few vascular points, or some trifling change, (often only an effect, an accident, an epi-phenomenon) with all the onus, and blame of causing many diseases which our fore-fathers, perhaps less subtle anatomists, but infinitely better logicians than we, denominated " general " or " morbi totius substantiæ "!

---

### XXIX. Fissures and Depressions observed on the Bones of the Cranium of New-born Infants after Natural Labours.

The occasional occurrence of these ought to be well remembered by medical jurists. Many medical men of great eminence, as Haller, Rose, &c. have committed the serious error of invariably attributing to extraneous violence, every appearance of crack or other injuries to the cranial bones of fœtuses. Rœderer and Baudeloque have the merit of first proving the fallacy of such an opinion; and the best obstetrical writers of the present day agree with them. A most satisfactory case is reported by Professor Siebold in his journal. The labour was tedious and painful, and the child was born dead. A large bloody swelling was found over the parietal bone; and on exposing the bone, three distinct fissures traversed it in different directions. No instruments had been used; nor had the body of the child suffered any injury after birth.

---

### XXX. TOTAL MORTALITY IN FRANCE FROM CHOLERA.

It appears from an official report, that the total number (the military excepted) of those affected with cholera in France, from its first invasion at Calais, on the 15th March 1832, to the 1st of January 1833, is 230,000; and the number of deaths 95,000. The government expense is estimated at rather more than a million and a quarter of francs.

---

### XXXI. M. PIORRY ON PNEUMONIA HYPOSTATICA.

By this term, the author means inflammation of the lungs induced and kept up by the "engouement pulmonaire," or distention of the vessels about the roots of the lungs, or any other part which lies lowest, in consequence of the subsidence or gravitation of the blood to that part. It is well known to anatomists, that the posterior parts of the lungs are almost always found on dissection to have a deep purple venous colour, while the fore parts of the lobes are pale and speckled. This appearance of congestion is correctly attributed to the subsidence of the blood after death, to the most depending parts, and constitutes the "engouement cadaverique" of the French pathologists. Now, M. Piorry is of opinion, that in enfeebled states of the system, especially in aged people, a tendency to the same subsidence may take place during life, when the patients are long confined to the horizontal posture, and may thus, if it does not actually prove an exciting cause, at least much aggravate any inflammatory affection of the lungs;—eight illustrative cases are adduced; they occurred in old people admitted into the La Salpetrière as objects of charity. Most of them had no serious disease, save old age, at first; but mere confinement to bed appeared to bring on an obstinate cough and other pectoral symptoms. Auscultation easily discovered the seat of the malady; the dullness on percussion, and the absence of the respiratory murmur, with the consecutive râles, heard on each side of the spine, shewed that it was the posterior parts of the lungs which were chiefly affected; and the post-mortem examination confirmed in every case the accuracy of the diagnosis.

Professor Boyer long ago remarked that it was most injudicious to confine old people for any great length of time to bed; and even when it is absolutely necessary, as in fractures, or other severe injuries, they should be told to sit up occasionally in bed, and thus prevent that congestion of blood, not only in the skin of the back, but also in the depending parts of the internal viscera.

M. Laennec had observed the frequency of pulmonary symptoms supervening in the course of malignant or typhoid enteritis; and added that "the pneumonias which occur in the course of malignant fevers are almost always latent, and that nothing is more common, especially in Winter, than this complication." Professor Fouquier used to attach great consequence to the examination of the thorax behind, in all typhoid enterites.

M. Piorry has observed that hypostatic pneumonias are not unfrequent in patients affected with dilatation of the heart, especially when there is contraction of any of the orifices, and indeed in all cases, where the circulation of the blood is in any way obstructed or embarrassed; for, under such circumstances, the gravity of the fluid is permitted to act more efficiently, and thus a tendency to engouement from subsidence, or hypostasis arises. Even in a state of perfect health, the horizontal posture will occasion a certain degree of this congestion; for it is found that on awaking from a long sleep, the back parts of the chest have less resonance on percussion, and give out a more imperfect respiratory murmur than at other times. Avenbrugger alludes to the dullness behind, in eruptive fevers, before depletory measures are employed. Piorry has noticed the same in many cases of fever and of angina; and the speedy removal of it, by bleeding and change of posture.—*Transact. Medic.*

The preceding views are sufficiently ingenious; but require further proof before we admit their entire accuracy. A simple and satisfactory test which the author has not employed, would be to place the bodies of his patients on their faces, immediately after death. The "engouement cadaverique" would be thus prevented, and the simple uncombined appearances from the supposed hypostatic pneumonia would be easily judged of.—*Ed.*

---

### XXXII. Purulent Diathesis—Pericarditis.

There is, in some states of the constitution, so great a tendency to the secretion of pus, or as the French have called it, "travail pyogenique," in all inflammatory attacks, that we may with reason say that there is a purulent diathesis. It is of great consequence to be on our guard in the treatment of such cases; for they will not bear active depletion well; two or three good examples of pericarditis very rapidly terminating in sero-purulent effusion are adduced in illustration. The danger is the greater, because the local or characteristic symptoms of the pericarditis are sometimes exceedingly obscure and indecisive. In one case of a girl convalescent from scarlatina, and in whom œdema had supervened, there were no symptoms of distressed breathing or of disturbed circulation, till within 2½ hours before death. On dissection, upward of 6 ozs. of reddish serum were found in the pericardium, and this membrane was lined copiously with lymph. A very interesting case of pericarditis with large effusion is mentioned, in which the symptoms of suffocation and dreadful anguish at the heart came on with periodic regularity at the interval of 24, 12, and 4 hours; these paroxysms being followed by perfect ease and tranquillity. Such cases have actually been mistaken for malignant agues. A memorable instance of this error was committed by Cabanis in the case of the famous Mirabeau. Rostan carries the idea too far, when he says that the paroxyms of all true asthmas are but the intermittent symptoms of diseased heart.—*Transact. Medicales.*

---

### XXXIII. Anatomical Anomalies.

No. 1. In an infant which lived fourteen days, and exhibited no signs of cyanosis, not only was the foramen ovale largely open, but the pulmonary artery, after having given off its branches to the lungs, curved round to the left side, and was continued down along the vertebral column in the place of the descending aorta, which was awanting. The aorta arose as usual from the left ventricle, and ascended towards the neck, where it bifurcated.

No. 2. The veins on the anterior walls of the abdomen were found enormously enlarged and varicose, forming on each side of the linea alba two immense pyramidal tumors. There existed probably some obstruction in the vena cava; and nature thus endeavoured to compensate, by enlarging the anastomosing veins between the iliac and femoral veins on the one hand of the vena portæ, and umbilical vein [which was not obliterated] on the other.—Lieutaud and Manec have reported similar cases of a magnified communication between the iliac and portal veins, and it is curious that this is the normal arrangement of the vessels in many reptiles.—*Ibid.*

### XXXIV. PLICA POLONICA.

M. Sedillot, who has lately returned from a sojourn in Poland, communicated to the Anatomical Society of Paris the personal observations he had made on this singular disease. One of the hairs taken from a plaited mesh was subjected to a microscope; by means of which a median canal gradually enlarging towards the free extremity of the hair was clearly seen. This canal was lined with a most delicate reticulated tissue, and in this tissue was contained the colouring matter; the bulb was distended and softened, and drops of matter could be squeezed from it. The disease commences in the bulbs, and propagates itself towards the loose ends of the hairs. After a time, the diseased secretion becomes less and less, and finally ceases, and the hair returns to its normal state.

### XXXV. OPEN FORAMEN OVALE, WITHOUT CYANOSIS.

In a child of seven years, who had never had any symptoms of the blue disease, the inter-auricular septum presented at the site of the foramen ovale a network of fibres, between whose meshes the blood might freely pass.

### XXXVI. PUS FOUND WITHIN FIBRINOUS CONCRETIONS OF THE HEART.

Two cases are adduced; one occurred in a syphilitic phthisical patient, the other in the body of an old woman, who was affected with asthma.

Tubercles were observed in the parenchyma of the heart in a patient who died of tubercular phthisis.

### XXXVII. PHLEBITIS OF THE CAPILLARY VEINS.

M. Ribes has repeatedly observed this in scorbutic old people at the Hospital of Invalids. The inflamed minute veins form painful and tense subcutaneous networks. The larger veins are sometimes affected simultaneously, and sometimes not.

### XXXVIII. INTESTINAL ENTOZOA.

There are four species, viz. ascaris lumbricoides, oxyurus, tricocephalus, and tænia; their mode of development is not well understood; they are either spontaneously engendered, or their germs are somehow or other admitted into the intestines, and there they grow and are unfolded. Their remote cause is probably always imperfect assimilation of the food. Each species is confined to a limited locality in the intestines. Whenever they are found in the air-passages, biliary or genito-urinary organs, we believe that they have escaped from the bowels.

The worms found occasionally in the cavity of the peritoneum also, and in fæcal abscesses, make their way from the bowels; but the perforations through which they pass are the result of previous ulceration, and are not caused by the animals. Such are the statements of Cruveilhier, a high authority in these matters.

### XXXIX. LOSS OF MEMORY AND OF SIGHT AFTER A GUN-SHOT WOUND IN THE SUPRACILIARY REGION.

An officer of the 30th regiment was struck by a ball, which lodged in the right frontal sinus. Amaurosis, as is common enough, of the right eye, was the consequence, and almost total loss of the memory of events and objects. *Vide Lancette Française—Phrenologia quid dicis?*

### XL. AMAUROSIS, FROM ONANISM IN A FEMALE.

A prostitute was admitted into the ophthalmic wards of the Hôtel Dieu, with great weakness of sight, amounting almost to amaurosis. She confessed that she was in the habit of polluting herself, and that she was immediately seized with complete blindness, whenever she addicted herself to the practice. It is stated that unfortunately such cases as the preceding are not very unfrequent among the young people of the seminaries and colleges in France!!—*Journ. Hebdom.*

XLI. Ossification of the Retina.

An example of this rare pathological phenomenon was found in the eye of an old woman who died lately at the La Salpetrière. The eye had been long atrophied. The retina, or rather the serous lamella, between the true retina and the choroid, had become the seat of an osseous deposit, which very much resembled in appearance the diploe in the cranium of birds, the cellular texture being very spongy and open. The vitreous humor had been greatly wasted.

M. Rognetta has detailed a case similar to the above; and it is worthy of remark, that ossification of the retina is not uncommon in horses, when their eyes have become atrophied from what farriers call periodic flux.

XLII. Aromatic Odour exhaled from the Fore-arm.

A man, aged 30, quite healthy, was surprised one evening after a day's active exercise, to perceive, when he undressed himself, a sweet strong-smelling odour, like that of Peruvian balsam, or the vapour of amber or of benzoin, from the inner surface of the left forearm, near to the wrist. He affirmed that he had not touched any of these articles nor any similar substances; but yet the smell was so powerful, that Dr. Speranza [who reports the case] was convinced that he had secreted some odorous matter about him. Friction and various lotions were used to counteract this curious symptom; but with no effect; indeed the rubbing, or any cause which heated the surface, seemed to encrease it. It did not come and go, but remained constantly at all times of the day;—and no other part of the body exhaled any particular odour. Not only Dr. S. but a number of other medical men examined this patient, and all were satisfied of the truth of the above statements, although none could give any satisfactory explanation. This very singular occurrence continued for two months; the patient fell sick of fever; and from the first appearance of the symptoms the odour ceased, and never returned, although the patient speedily regained his health.—*Annali Universali, Feb.* 1832.

XLIII. Chloride of Lime, as a Lotion, against the Itch.

Professor Fantonetti gives the result of nine trials. In seven of these cases the disease was cured in from six to eight days; in the 8th case the psorous eruption was removed, but was followed by an eczema, which gave way under the use of tepid baths; and he left the hospital well; the itch however re-appeared, and then yielded, only to sulphureous fumigations. In the 9th, the psora was cured—re-appeared—was cured again—returned a third time—and was finally subdued by the sulphureous fumigations. The chloride of lime lotion is made, by adding $1\frac{1}{2}$ or 2 ounces of the chloride to a pint of water; it ought to be rubbed three or four times a day on the affected parts. A simple tepid bath is to be used every third day, in order to soften the skin, and clear it of any calcareous crusts. If the lotion is too irritating, its strength must be reduced. The disease is generally cured effectually in eight days.—*Ibid. Sept.*

XLIV. Fatal Effects of a Tartar-Emetic Plaister.

M. Bricheteau, physician of the hospital Necker, reports the case of a girl aged 20, for whom a plaister, the surface of which was sprinkled with half a drachm of tartar-emetic, was ordered to be applied to the epigastrium, where several fresh leech-bites were at the time. In the course of two days a deep eschar was formed; the subjacent cellular tissue rapidly destroyed, and the recti muscles made bare; much febrile irritation was excited, aphthæ appeared in the mouth, and the parotids became immensely swelled; the patient died. On dissection, the whole of the cavity of the mouth was found studded with aphthæ; the inner surface of the small gut presented considerable redness and puffiness; and the ulcer in the epigastrium extended deep to the posterior surface of the recti muscles.

*Observations.* Blisters have frequently been known to cause painful and most troublesome œdema; and leech-bites have been followed by erysipelas and deep ulcerations. As a general remark, epispastics must be employed with caution, in weak, irritable, and lymphatic females.—*Archiv. Gen.*

### XLV. DEATH FROM THE HÆMORRHAGE OF LEECH-BITES.

A young female had twelve leeches applied to the abdomen. The blood had continued to ooze all night, and on the next day she was found bloodless and exhausted, and, in spite of all the means used to revive her, sunk. On dissection, all the viscera, especially the heart and liver, were found remarkably pale. To ascertain the probable quantity of blood lost, M. Bricheteau applied a small wine-glass to a leech-bite, which at the time was bleeding moderately; in ten minutes he collected 3 drachms;—if the discharge continues, more than 2ozs. may therefore be lost in an hour, and 48ozs. or 3lbs. in 24 hours, from one leech-bite.—*Ibid.*

### XLVI. KERMES MINERAL IN PNEUMONIA.

This preparation of antimony, the hydro-sulphuretum rubrum, may be advantageously substituted for the tartar-emetic. In one case reported by Bricheteau, six grains were given the first day, and copious purging was induced; the dose was gradually increased, so that on the fourth day the patient took 15 grains;—the "tolerance" was induced on the second day. The result was quite satisfactory.—*Ibid.*

### XLVII. M. PIGEAUX ON THE CAUSE OF THE SOUNDS OF THE HEART, AND HIS REFUTATION OF MAJENDIE'S THEORY.

This is a very valuable paper, and will amply repay the study of all aucultators. We regret that we cannot afford room for a more extended analysis. M. P. says that he was first led to doubt the accuracy of Laennec's explanation on acoustic principles. He found that no contraction, however quick and energetic, of the hand when plunged in any liquid, ever produced any sound; but that if, on the contrary, he jerked a small jet of water against the walls of a metallic vessel, sounds, varying in intensity and timbre, and somewhat akin to those of the heart, were caused; again, when he tied the neck of a bladder filled with water to one of the orifices of the heart, and squeezed the bladder in jerks, he could still more closely imitate the normal sounds of the heart. He performed many experiments on frogs, lizards, and snakes, and satisfied himself that the succession of the systole and diastole, both of the auricles and of the ventricles, is uninterrupted, and therefore that there is no appreciable period of repose; that the ventricles only, and not the auricles, empty themselves completely during their systole; and that the contraction and dilatation of the auricles are performed less quickly than the corresponding action of the ventricles. Pathology taught him that the morbid sounds, viz. the bellows, saw, and rasp sounds, are caused by the vibrations of the valves, in consequence of the friction of the stream of blood, and that all the modifications of the clear sound are invariably associated with certain changes in the state of the large vessels.

The following propositions contain a summary of his opinions. 1. The circulating fluid is the immediate cause of the sounds, which have hitherto been attributed to the contraction of the cavities of the heart. 2. The shock or friction of the blood against the parietes of the vessels which it permeates, occasions vibrations, which give rise to the sounds. 3. The intensity of the sounds is proportional to the force of this impulsion; the organization of the parts which enter into vibration determines the timbre or tone. 4. The contractions of the cavities of the heart are only the mediate cause of this phenomenon, which is also coincident with, but not occasioned by the dilatation of these cavities. 5. The movements of the heart, considered by themselves, are quite "aphonic;"—the fact of the interposed periods of silence between the different sounds is a proof of it. 6. When the blood enters the auricles, it dilates them without any murmur. 7. Driven into the ventricles, by the equally noiseless contraction of the auricles, the blood impinges upon the walls of a cavity, which, closed at its exit, enters into vibrations, and occasions the first or dull sound, or, as it ought rather to be called, the inferior sound. 8. The time of the first repose, or rather of the first silence, is the instant of the systole of the ventricles. 9. The second, clear, superior, or upper sound follows immediately, and is occasioned by the collision of the blood against the walls of the aorta and of the pulmonary artery. 10. The period of the second silence, or as it is called by Laennec, the period of the repose of the heart, is equal to the difference of the time which is occupied by the clear sound on the one hand, and by the dilatation and contraction which are aphonic of the auricles on the other.—[This last proposition is very obscure—Ed.]

The theory of M. Majendie is very different from the preceding; he maintains, in a memoir recently read before the College of France, that the two sounds of the heart are owing to the successive impulsions of the apex and of the base of the heart against the walls of the chest; that the diastole and dull sound are synchronous, and that the systole and clear sound are also synchronous; and that if fluid be artificially injected into the pleuræ, and thus prevent the shocks of the heart against the ribs, the sounds can be no longer heard. But this theory is at once proved to be insufficient and erroneous, by the fact that the sounds of the heart can be heard distinctly, after the sternum and ribs have been removed. Numerous experiments set this beyond doubt. Moreover, no explanation can be given of the morbid bruits or murmurs, on the above theory. In all our enquiries on the cause of the cardiac sounds, it is of great importance, says M. Pigeaux, to remember that the exact points of the maximum of intensity of each are separated, or apart about three inches from each other.—*Arch. Gener. Nov.*

---

### XLVIII. Neuralgia.

A severe case of supra-orbital neuralgia is mentioned, in which quinine combined with acetate of morphia, very speedily and decidedly effected a cure. —*Journal Complementaire.*

---

### XLIX. Intermittent Spasmodic Cough.

An instructive case is recorded by M. Bricheteau, in Nov. No. of the Journal Complementaire. The ordinary treatment, by leeches, blisters, emetics and so forth, had failed; and an injection composed of 10 grains of assafœtida, 3 grains of quinine, a yolk of an egg, and 6 ozs. of water, was given with the happiest effects.—*Ibid.*

---

### L. Dupuytren's Mode of treating Prolapsus of the Rectum.

This eminent surgeon has cured a vast number of patients labouring under this disease by means of an operation which is both simple and speedily effectual. He seizes the folds around the anus with forceps whose blades are large and flat, and excises them with strong sharp scissors; this excision must be carried deep enough to remove not only the integuments embraced by the forceps, but a portion of the rectum, when the relaxation of its mucous coat is very considerable; generally the depth of the wound need not exceed a few lines, but in other cases it must be at least an inch. Dupuytren usually cuts off in this manner four folds of the margin of the anus, one in front, one behind, and then one on each side; if the disease be not of great extent, the removal of one or two of the folds may be sufficient. This operation is seldom followed by any considerable hæmorrhage. It is necessary that the patient be so treated, that he has no occasion to have the bowels relieved for eight or nine days, in order that the wounds may not be disturbed. The cure is generally complete in a fortnight. Dupuytren has met with no case of failure hitherto.—*Ibid.*

---

### LI. RESEARCHES ON THE CONIUM MACULATUM, BY PROFESSOR FODÉRÉ.

It has been frequently disputed, whether it was really this plant which the Athenians used for state poisoning, as in the case of Socrates. The description left us, in the dialogue of Plato with Echecrates, who was present during the last hours of the sage, is as follows :—after drinking the fatal cup, he kept walking about the room for some time, till he felt his limbs grow heavy; he then lay down on his back; the executioner went up to him and pinched his feet, asking him whether he was sensible of it. Socrates answered, "no." The numbness and loss of feeling extended up the legs and thighs, which became gradually stiffened and cold; the belly was soon similarly affected, and when the coldness reached the heart, life was immediately extinguished. Just before his death, he took off the cap from his head, and said to Crito—"We owe a cock to Esculapius, forget not to discharge the debt." Shortly after these words he was convulsed; his features became fixed, and the dismal scene was closed.

Now, no medical man could very satisfactorily assert, that the preceding description could lead him to ascribe the poisoning to the common hemlock; and M. Fodéré, till very lately, confessed that it must be considered as very uncertain, what was the herb which had been used. His recent experiments have however satisfied his mind that it was in truth the conium. The beautiful memoir of M. Geiger of Heidelberg, in 1832, has thrown much light upon our inquiries. He has found that the active principle, which he denominates cicutine, is an alkaloid, in itself volatile, but fixed in the plant by a peculiar acid; that it is of an oily consistence, has a sharp, very penetrating and offensive smell, resembling that of a noxious urine, is acrid to the taste, like tobacco, and is soluble in water, alcohol, and æther; that besides this principle there is another, equally volatile, having the ordinary smell of the plant, but which is perfectly innocuous; that the cicutine can be obtained only from the fresh plant; and that it becomes decomposed even in the extract prepared from the recent plant, after six weeks' keeping; that a third of a drop killed a pigeon, and that a dog, to which eight drops had been given, soon began to totter, then fell down, vomited, and died in about six minutes, after being violently convulsed.

Professor Fodéré has repeated M. Geiger's experiments, and has simplified considerably the process to obtain the active principle of the poison; he finds that it exists in the plant, as a subconiate of cicutine. We cannot afford space to give the particulars, and therefore recommend to such of our readers as are interested in pharmaceutical operations, to consult the paper itself in the 173d number of the Journal Complementaire des Sciences Medicales. He gave six grains of the cicutine to two strong rabbits—they soon began to totter, the pupils became dilated; they yawned and fell into a deep sleep; in about half an hour they awoke, and seemed to have recovered from the effects of the poison. These results prove that cicutine has sedative and narcotic qualities nearly equal in activity to those of morphine, but very inferior to those of strychnine, two grains of which are sufficient to kill a rabbit very speedily, whereas ten and even 15 grains of acetate of morphine have been given, and stupor only was induced. To try the effects of a full dose of cicutine, Fodéré gave one rabbit twenty grains; the animal became immediately convulsed, seized with tetanus, and this was quickly followed by a general stiffness; the pupils were at first dilated and then contracted; death took place in the course of two minutes. Now by attending to these phenomena we can easily trace the analogy between them, and the circumstances of the death of Socrates. The numbness and stiffness, mentioned in Plato's dialogue, were doubtless the tetanic rigidity observed in the above experiments, and the stupefaction of all the senses and functions, the fixed look, and the convulsions, correspond in both cases; and if we were to regard the last injunction of the sage respecting the sacrifice to Esculapius, as the ravings of delirium, we should probably be right, especially as his condemnation was in fact awarded, for the very opposition he had always shewn to the superstitions of his country. If hemlock was indeed the poison employed, the dose must have been considerable; probably eight ozs. at least of the juice.

We shall only advert to one topic more: it has been already observed that the cicutine could be obtained from the fresh plant, but not from dried specimens, nor from old extracts. This shews the importance of preferring the recent herb on all occasions, when we wish to employ conium medicinally. So inert is the extract sometimes, that it has been given in the dose of an oz., without producing any very obvious effects; but as we cannot always have the fresh plant, it is an object of great consequence to contrive a plan by which a really active preparation may be had and preserved. Fodéré suggests that the method pursued by many of the German chemists is the best; namely, by preparing an extract by means of an hydraulic press; and he has already ascertained, that when so procured, it retains perfectly the peculiar smell of the fresh plant.—*Ibid.*

### LII. Hydro-ferro-cyanate of Quinine in Intermittent Diseases.

Dr. Cerioli adverts to the frequency of the relapse of agues, after their apparent cure by the ordinary preparations of cinchona, and points out the necessity of attending to the existing visceral derangements during the course of treatment; for it is true that we may succeed in removing the periodic fever by means of bark alone; but to effect a permanent cure, remedies must be simultaneously directed against any pathological lesion of the liver, spleen, or any other viscus which may chance to be affected. Hence, in numerous cases, it is requisite to combine an antiphlogistic with an anti-periodic therapœia. Like most Italian physicians, Dr. C. regards the hydrocyanic acid as a powerful sedative of inflammatory action, and he was, therefore, led to expect beneficial effects from exhibiting it along with bark in many intermittent diseases. An extended experience has substantiated the correctness of his views, and proves the great utility of the practice in all agues which are kept up, or are increased by, visceral irritation.

He has published the results of 24 cases, in all of which the quinine alone had been used without effect. In 13 of these the type was quartan, and enlargement, or sub-acute inflammation of the spleen, existed; they were chiefly old and obstinate cases, of from a few months to one, three, and even eight years' standing. General and local bleedings, resolvents, and bark, had been freely administered. The hydro-ferro-cyanate of quinine was given, in the dose of two, three, four, and eight grains, made into pills, in the course of the day; sometimes it required still larger doses. In other cases, there was chronic hepatitis, general debility, anorexia, or gastric irritation. Repeated leechings, emollient enemata, mercury, a low diet, and tepid bathings, generally subdued these symptoms; but the ague still continued, in spite of the use of the quinine. To counteract a new congestion of the liver, or of the stomach, Dr. C. gave the hydro-ferro-cyanate with complete success. He very properly inculcates the necessity of first subduing the local disease, before we can expect to cure the ague with bark in any form, and only asserts that, when the sulphate of quinine has failed (the extent to which it was carried is not mentioned), he has often succeeded with the new preparation. An interesting case of intermittent sciatica is reported, and also three cases of pulmonary irritation and hæmoptysis, attended with an aguish fever, in all of which the hydro-ferro-cyanate was most beneficially substituted for the sulphate.—*Annali Universali, July.*

### LIII. Efficacy of Iron against Chlorotic Gastralgia.

MM. Bonnet and Trousseau have reported several interesting cases, in which severe and obstinate pains in the stomach, occurring in females affected with amenorrhœa, leucorrhœa, or similar uterine affections, have yielded to the judicious employment of subcarbonate of iron, after most other means of treatment have failed. As the first case is a good illustrative specimen of the author's views, we shall give an abridged statement of it.

A. B. aged 21. She had enjoyed almost uninterrupted good health till her sixteenth year, when the catamenia first appeared. Since that period, she has been sometimes regular, sometimes not so, and has suffered much from headaches and general indisposition. In the beginning of 1829, she first experienced symptoms of gastralgia; the pain she compared to a weight, and sometimes to a pinching—it was always worse after eating, especially animal food. At first it was not very severe, and came on only at intervals of several days; but gradually it became worse, and returned almost every day or two. A physician prescribed leeching of the epigastrium and demulcent drinks—flesh, fruits, and vegetables were permitted, but wine and coffee interdicted. The distress was relieved for some time under this treatment, but, in the course of a month or so, it returned with aggravated violence; at last she suffered so much after every repast, and even after a single spoonful of food, that she almost starved herself. The physician considered that there was chronic gastritis, and even suspected cancer of the pylorus. The patient was exceedingly wasted, but retained her wonted cheerfulness. MM. Bonnet and Trousseau were consulted at this period; they enquired what description of food seemed to cause least pain; she told them boiled or stewed fruits, prunes, &c. but that all such articles had been inhibited by her medical attendant. She was now permitted to take them, and the following pills ordered: take of carbonate of iron, extract of succory, equal parts, and divide into pills of six grains each; the dose was at first only one night and morning, and gradually increased to eight or ten at a time. In 20 days she was almost completely cured. The iron was continued for two or three months.—*Archiv. Gener.*

### LIV. Singular Convulsive Disease.

A young man, aged 22, consulted, in May, 1825, M. Husson, physician of the Hôtel Dieu, for a very extraordinary convulsive disease, which had afflicted him for several years. M. H. called in the evening upon him, and found him apparently quite well, and enjoying the conversation of a friend; on a sudden, he began to move about in his chair, struck his right thigh forcibly with his right hand, which he then immediately placed on his left shoulder; then, with his left hand, he struck his left thigh, and placed it on his right shoulder—the two arms thus crossing each other on the chest. There were thus four slaps, following with admirable regularity, first on the right thigh, next on the left, then on the left shoulder, and, lastly, on the right one; and so quickly were they done, that it was difficult to follow them with the eye; in about 2½ minutes those movements abated and ceased for a second or two; then, after a very deep inspiration, they returned for about a minute and a half, and again ceased; but, after another deep inspiration, the movements were repeated a third time, but did not continue so long; the paroxysm altogether lasted for about five minutes; the patient then rose from his chair, walked briskly about the room, stretched out his arms as if to unstiffen them, and resumed his former health. The paroxysm, it will be observed, consisted of three distinct attacks, separated by short intervals, and becoming less severe each time. During the continuance of it, the patient felt slight vertigo, but did not lose his consciousness; he could hear noises, but he could not answer any question, and afterwards he had only a confused recollection of what he had seen or heard during the fit; his features were fixed, but not convulsed. These fits had repeatedly seized him in public places, and then he was considered either as an idiot or impostor. In 1823, when he was seventeen years old, he had laboured under typhus fever, and had been delirious for nine days. It was while recovering from this fever that the first appearance of the convulsive disease was noticed; but at that time it assumed a different type, and much resembled a fit of epilepsy, as the patient was quite insensible, and foamed at the mouth. After a lapse of four months, it took on its present very extraordinary character. At first, there were three paroxysms in 24 hours—then a fourth was added, in consequence, it seemed, of mental anxiety and grief; and, for $2\frac{1}{2}$ months, there were four paroxysms regularly every day, namely, when the patient rose from bed—at 10 o'clock—at noon—and, lastly, at the exact time of sunset. The 10 o'clock paroxysm ceased spontaneously, and the noon one after a long journey which the patient made a-foot; so that, when M. Husson was consulted, there was only a morning and evening attack; and, what was very singular, these attacks came on precisely when the patient got out of bed and when the sun set, following the variations of the time. Another very curious feature is, that the number of slaps or thumps which he gave himself was always the same, in the corresponding paroxysm: in the morning one, 110 were given to each thigh, without any interruption—in the evening one, which, as we have seen, was composed of three attacks, 110 were given in the first, 60 to 65 in the second, and 30 to 35 in the third one. These minutiæ may seem trifling to some, and may, perhaps, not be readily believed; but it is right to state, that not only M. Husson, but also MM. Esquirol, Villermé, and the late M. Dance, repeatedly have authenticated the preceding details. Generally, before each paroxysm, the patient perceived a sort of "aura," which he compared to a shaking, from the point of the toes upwards to the shoulders, but not higher; whenever he felt this sensation, he immediately sat or lay down, else he fell to the ground. Depletion, baths, antispasmodics, narcotics, tonics, &c. had quite failed to relieve him. In spite of all the efforts of the Parisian physicians, the disease remained untouched, and the patient returned home.

*Remarks.* Professor Frank has reported the case of an epileptic girl, in whom the fits returned regularly with every new quarter of the moon: "Et quidem tam exacto calculo, ut ipso momento quadræ lunaris, et inscia sæpe penitus hujus mutationis in cœlo, rhythmum lunarem in suis paroxysmis explicaret."—*Archiv. Gener. Sept.*

### LV. Indian Ophthalmia, treated with much success by Alum.

M. Sonty, in a report which he lately made to the Minister of the French Marine, mentions his great success in the treatment of a most violent and rapidly-destructive epidemic, purulent ophthalmia, in the East Indies. At first he had employed antiphlogistic measures, but they entirely failed, or rather the disease was too intense to be quickly enough affected by them. The natives employed very stimulating applications; as, for example, a mixture of pepper, lemon-juice, and the juice of tamarind leaves, to which is added afterwards roasted gall-nuts; this paste they applied round the eyelids. M. Sonty soon found out the marvellously good effects of rock alum. He took a piece, with which he kept stirring, for eight or ten minutes, the white of an egg, which is then to be put into a fine muslin bag. When this is to be used, the patient's head must be held back, and, while the eyelids are kept open, a few drops of the liquid are to be squeezed from the bag upon the eye; this operation must be repeated very frequently—in some cases every half-hour. The same treatment is applicable in all the stages of the disease, and generally cures it in from 24 to 48 hours.—*Archiv. Gener.*

---

### LVI. M. CLOT BEY, ON THE CHOLERA IN EGYPT.

There is a very interesting and instructive paper by M. Clot in the September No. of Broussais' *Annales de la Medecine Physiologique;* and such of our readers as are anxious to become acquainted with the history and travels of this pestilence, will do well to study it. We can afford only a very short space for a few of its most important details. In June of 1831, it was first announced at Cairo that cholera had broken out at Mecca; and by the 15th of the following August it had spread to Cairo itself. The terror and desolation which speedily followed were in truth awful. The mortality exceeded that of the most fatal epidemics, and threatened utterly to sweep away the population of Cairo. The disease was so intensely violent, and the "nullity" of the treatment so complete, that scarce one recovered from the attack. At almost every step we saw some of the wretched victims suddenly arrested by the pestilence, fall to the ground as by the breath of a destroying angel, and expire after one or two hours' sufferings; there was no hospital but one, and that was for the military exclusively; and as for homes they had none. Lying in the streets, they died unassisted; even a drink of water to assuage their intolerable thirst was seldom to be had. The Moslem resignation, which had never been shaken by the most frightful pestilences, gave way for the first time; and the Turks, following the example of their Pachas, fled from the place. The symptoms, as detailed by M. Clot, are quite the same as we have seen in aggravated cases at home; but in Egypt, as here, there were various shades and degrees of virulence, and few or none escaped it altogether. In the more favourable cases there was only loss of appetite, nausea, slight vomiting and purging.

M. Clot is a decided Broussais-ist in treatment: if called sufficiently early he bled, ordered warm drinks, sinapisms, frictions, and administered opium freely. If there was much pain at the epigastrium, he applied leeches or the cupping-glasses. In some cases he repeated the venesection a second and even a third time. If the patient was already in the cold stage, bleeding was useless, and indeed impracticable, for the blood would not flow; the indication was to restore the cutaneous circulation, and for this purpose sinapisms, rubbing, warm clothing, and drinks, to which was added some narcotic, were prescribed. If the heat returned to the skin, venesection was immediately performed. In general, the antiphlogistic treatment, pursued actively from the commencement, was the only one whose efficacy, comparatively, was constant. M. Clot has saved many, he thinks, by it from the very worst state; and so satisfied were the people of its superior safety, that they practised it on themselves, on the first appearance of the symptoms. A lieut.-colonel of a regiment, whose surgeon had disgracefully fled, saved almost all the soldiers attacked, by bleeding them himself. During the prevalence of the scourge the sky was gloomy, ominous, and obscured by a grey veil; the sun gave a pale and sickly light, which became of a green hue towards sun-set; and this was followed by a long twilight, whose reddish glare lasted for several hours.

On the interesting topic, of the mode of the propagation of the disease, M. Clot comes to the conclusion, that it is not by contagion, but through the medium of the atmosphere. His reasons are so just and forcible that they deserve to be recorded. 1. The disease broke out at nearly the same time over the whole of Egypt; and therefore appeared in many places which had no communication with Haggiaz, where the first case was seen. 2. The Harams, watched with the most strict quarantine, were not exempt. 3. The sailors in many of the ships in the roadstead of Alexandria were seized, although no communication had taken place with the shore. The Vice-roy of Egypt had embarked on board a vessel on the first outbreak of the disease; but as several of his retinue successively died, he had to change the vessel several times. 4. Five hundred Bedouins, encamped in the desert, many miles from Abouzabel, and separated by a most strict quarantine, did not escape. 5. Some villages remained altogether free, although there was a constant communication between them and infected places. 6. There was great inequality in the mortality of different places, under nearly similar circumstances. 7. Very few of the servants of the hospitals were seized. 8. The bazaars, which had been closed at first, were re-opened, when the disease had nearly subsided, for the sale of the clothes and other effects of those who had died; and although no precautions were used, the disease did not re-appear. 9. The frightful rapidity with which the disease reached its acmé of destructiveness. Can we suppose that a disease, if merely contagious, should have, in the course of four or five days, acquired its summum of intensity; and after lasting for scarcely a month, should as quickly subside? 10. The almost universal dissemination of the disease in some of its forms, from the living death of the malignant, to the slight purging in the mild cases. M. Clot, although a decided anti-contagionist, admits that cholera, like other epidemics, may, under certain circumstances, acquire a contagious character.—*Annales de la Medecine.*

---

### LVII. On Cyanosis.

We employ the word in its original and comprehensive acceptation, to denote that morbid state in which the skin and mucous membranes acquire a blue or purplish hue.

M. Gintrac published a memoir on this subject in 1814; Professor Frank, of Wilna, has devoted a chapter of his "Praxeos Medicæ Universæ præcepta" to consider the "morbus cæruleus;" and, lastly, Jourdan has written an able article on "Cyanodermie" in the Dictionnaire Agrégé des Sciences Médicales. Frank's definition is—"lividity of the skin, especially of the lips, hands, and feet; coldness of the extremities, irregular movements of the heart, dyspnœa, returning at intervals, weakness of the muscles, and tendency to hæmorrhages from different parts." He divides cyanosis into the congenital and the acquired forms. In the former, the new-born infant presents, after a general paleness of the skin, a livid, leaden, or a reddish hue of the surface, and this hue may alternately fade and re-appear for some time. The lividity is always increased during the act of sucking, or when the child cries, coughs, or is kept upright. The dyspnœa and cough are sometimes constant—at other times intermittent; the paroxysms are usually accompanied with convulsions, and after these the little one lies almost inanimate. During this time, although the heart beats violently, the pulse is frequent, but regular, and the other functions are normally performed.

In the acquired form of cyanosis, the lividity is first noticed in the nails, points of the fingers and toes, and in the lips; the dyspnœa recurs at intervals, and is broken with sighing and groans—the action of the heart and arteries is violent and irregular, and sometimes the jugular veins are enormously distended. After these paroxysms, the breathing becomes easy, but the patient complains of a continual coldness, and yet this is, perhaps, not appreciable by another person, nor by the thermometer. If dentition and puberty are but slowly developed, the body grows rather quickly, the arms are long, the last joints of the fingers are puffed and swollen, rough and warty, the nails curved inwards; the face is slightly swelled, the eyes are prominent and yellowish, the gums spongy, the breath offensive, the tongue is unusually large and irregular, and the patient is often yawning—sometimes the skin is very tender in particular parts; the sleep is generally disturbed by fearful dreams, yet the patient's powers are not dull, nor enervated; the appetite may be good, but the patient's distress is always aggravated during digestion, and also by moral emotion, by cold, and any catarrhal ailment. There is a great tendency to bleedings from the nose, gums and lungs. As the patient becomes older, he is annoyed with headache, vertigo, dimness of vision, a sense of anxiety and pain in the chest, nausea, and general debility; the bowels are constipated, and the urine occasionally thick and fetid. There is generally a paroxysm every morning and evening, and it lasts from a quarter to half an hour; when it is coming on, the patient lays himself upon his belly, and compresses his chest with both his hands. If he does not die suffocated in one of these fits, dropsy, hemiplegia, or hemoptysis, very frequently brings on the fatal issue.

Frank considers hereditary tendency, the male sex, and perhaps, also, any fright of the mother during gestation, among the causes which predispose to cyanosis.

If the disease be not congenital, it may first appear at very different periods of life—sometimes in a few days after birth, or in the second week—in the first, second, or sixth month—towards the end of the first, or during the second, third, fourth, fifth, twenty-first, or twenty-third year, and at other times not till the fifty-seventh year. The exciting causes are usually dentition, the milk of an unhealthy woman, hooping-cough, variola, catarrh, amenorrhœa, blows on the chest, falls from any height, quick running, mental emotions, and cutting the hair in plica polonica. The proximate cause is either the mixing of the venous with the arterial blood, in consequence of some malformation of the heart and great vessels, or some impediment to the change of the venous into arterialized blood, as from the obliteration of the pulmonary artery, or from its being given off by the left and the aorta by the right ventricle, or from some obstruction to the action of the lungs, or, lastly, from an affection of the brain and spinal marrow. It is very common to find, on dissection, several of these morbid states conjointly; thus, narrowing of the pulmonary artery is often associated with, if it is not the direct cause of, the foramen ovale being open, or with a communication between the two ventricles. Our readers will now understand why Frank divides the disease into the cardiac, pulmonary, and the encephalic cyanosis. When the primitive seat has been in the lungs, preventing or impeding their action, it is called by him pulmonary; but in this form some morbid change either of the heart, or of the large vessels is very speedily induced; thus Lentin found enlargement of the heart, contraction of the great pulmonary vessels, and tuberculous hardening of the lungs together. Trotter found enlargement of the right auricle, and hydatids in the right ventricle with copious effusion in the chest and head. Heischman, in one case of cyanosis, found contraction of the tube of the trachea, and also of the glottis; and Kwiatowski mentions another, in which the only morbid appearance on dissection was hepatization of a large portion of the lungs. Still, as a general remark, we should say that not only are the lungs very frequently found dreadfully disorganized, and yet no symptoms of cyanosis are present, but, also, that their structure is often little or not at all diseased, in many cases of fatal cyanosis; and we are, therefore, inclined not to admit Frank's pulmonary species, regarding most of the cases which he adduces as rather belonging to the cardiac species, or that which is primarily connected with some malformation or disease of the heart. With regard to the encephalic form, we are bound in truth to recognize it; for numerous cases of well-marked cyanosis are on record, in which the heart, large vessels, and lungs, were found perfectly healthy, and the only morbid appearances existed within the head, or spinal canal. Such cases occasionally are met with, when the catamenia have been suddenly suppressed—in one instance which is mentioned nothing irregular was discovered, except a general contraction of the arterial system, and a very marked fulness or enlargement of the viscus over the whole body. A blue colour of the skin is, as is well known, induced by the internal use of the nitras argenti, and by certain poisons; it exists also in the cold stage of agues and of other fevers, and especially in cholera. Professor Broussais, in his history of the Phlegmasiæ, published in 1808, particularly alludes to the blue colour of the skin in some severe abdominal inflammations, and in his memoir on Cholera, recently published, he attributes the cyanosis of the surface to the stagnation of the blood induced by the propelling forces of the heart being much impaired, or even totally arrested; and the cause, according to him, of the moving power of the circulation being so much affected is a general inflammation of the gastro-intestinal mucous surface, and not any affection of the nervous system, nor any direct paralysis of the heart's action, nor any miasmatic or poisonous infection, nor yet any spontaneous and accidental alteration of the blood itself, for all these are consecutive and not primary phenomena in the disease. We are therefore to consider the arrest of the circulating and respiratory functions as subsequent to and dependent upon the violent affection of the bowels and also of the cerebro-spinal system; and that indeed any morbid condition of the intestinal canal, or of the uterus, or of the encephalon, may be attended with cyanosis, when any disturbance of the breathing or of the movement of the blood is induced. If we follow Frank's classification, such cases of the disease must be referred to his second species, viz. that which arises from some obstacle existing to the change of the venous into arterialized blood; but we ought to add to the number of his species, and admit at least other two; we shall thereby have the cardiac, pulmonary, encephalic, or cerebro-spinal, gastro-enteritic, and the uterine cyanosis, according as the primary seat of the disease is in the heart, lungs, nervous system, stomach and bowels, or in the womb.

It is an interesting fact, that many cases of cyanosis owe their origin to some disturbance of the menstrual function, especially to sudden suppression of the discharge.

Cyanosis, whether it be congenital or acquired, is very rarely cured. If the infant survive the first few days or weeks, it very frequently lives on to puberty; but an aggravation of the symptoms every now and then takes place, especially when any change is going on in the system, as at weaning, during dentition, or when the child begins to walk; so also at puberty; if this epoch be passed, the patient may live to 18, 20, 31, 40, 60, or even 80 years of age. The chances depend considerably on the nature of the organic disease, and of the accessory morbid states; thus, in a child whose pulmonary artery was found to be completely obstructed, death took place on the 13th day from its birth; whereas out of three patients in whom the foramen ovale and ductus arteriosus were simultaneously patescent, one lived 17 years, another 29 years, and the last 42 years. Male children have been observed to die more quickly than female ones; and the mortality is certainly greater in Winter than in Summer. It is quite unnecessary to allude to the treament of cyanosis; if the physiology of the disease be understood, it is easy to adapt our palliatives to the most urgent distress.—*Journ. Hebdom.* No. 113.

---

### LVIII. Cases of Pleuro-Pneumonia, with the Auscultatory signs in each, treated by large Bleedings.

Case 1. *Pleuro-pneumonia of the Right Side, in the second stage—great prostration.*

F. G. 58. When he was brought to the Hôpital de la Charité, so great was his exhaustion that he might have been supposed to be affected with malignant typhus, had not the sputa, which are so characteristic of acute inflammation of the lungs, and the signs obtained by exploring the chest, removed all uncertainty from our minds. The sputa were viscid, transparent, and of a rusty colour. Percussion over the fossa supra-spinata gave out a dull sound; and at that part no respiratory murmur could be heard; but along the base of the scapula and between its inferior angle and the spine, a bronchial, or blowing respiration, and a strong resonance of the voice, amounting almost to œgophony, were perceptible. Lower down, a crepitant râle of small bubbles was heard, and the sound was somewhat obscure. He had been bled the day before, and the blood was sizy. Ordered to be bled freely, and have 12 leeches to the chest. Next day (22d) the blood was found to be buffy and cupped; the patient felt relieved; auscultatory symptoms the same; pulse 110; respirations from 32 to 36. To be again largely bled, and the chest, when it is painful, to be cupped. On the 24th all the symptoms were much abated; and in spite of the active depletions, the patient felt stronger; the same dulness on percussion remaining; the resonance of the voice no longer accompanied with the œgophonic shrillness; bronchial respiration as before; crepitant râle heard very distinctly over the fossa infra-spinata, and under the inferior angle of the scapula; pulse 92; respirations 28. On the 26th percussion yielded a sound less dull; bronchial respiration and bronchophony remaining. The patient was now convalescent; but, although the returning crepitating râle, "rhonchus crepitans redux," extended itself considerably, the dull sound on percussion, and the bronchial breathing continued; in a few days he left the hospital well.

CASE 2. *Pleuro-Pneumonia of the Left Side, in the second stage.*

P. L., aged 30, entered the Hôpital de la Charité on the 21st of September. He had pyrexia, pain in the chest, cough, dyspnœa, and expectoration of transparent, glairy, and rust-coloured sputa. She was largely bled; and on the following day the report is, that the symptoms are still very urgent; breathing very hurried and laborious, 48 to 52 times in the minute, and at each act of inspiration the alæ nasi are violently lifted up; the cough is painful and frequent; the sputa rusty, gummy, and flowing out when the vessel is inclined, "en nappe." On percussion, the chest sounds well in front, but behind, on the left side, there is dulness over the whole of the fossa infra-spinata, and then no respiratory murmur is to be heard, but a bronchial blowing, or "souffle," and a jerking resonance of the voice. The prognosis was not favourable, as the disease had extended very rapidly in a short time, and the patient had suffered from dyspnœa for the preceding five years, in consequence of a neglected pneumonic attack. Ordered to be freely bled and leeched.

On the 23d she was much relieved; the breathing easier and not more frequent than 36 or 40 times in the minute; the sputa less streaked and coloured with blood. The exploration of the chest gave the same signs as yesterday. Venesection to be repeated, and blood to be drawn also by cupping over the fossa infra-spinata. On the 24th, respirations only 28 to 32 in the minute; sputa thin, mucous, and transparent; the bronchial souffle, bronchophony, and œgophony well marked. On the 25th the ear began to hear the "râle de retour" at several points. On the 26th patient not so well; the crepitating râle heard yesterday, no longer appreciable; the breathing and the pulse increased in frequency. Ordered to be bled. On the following day, the crepitating râle returned, and gradually, but slowly, the respiratory murmur was to be heard; the resonance of the voice also was heard faintly on the 4th or 5th of October; but the sound on percussion still remained very dull.

CASE 3. *Pleuro-pneumonia of the Left Side, in the second stage, accompanied with Typhoid Symptoms.*

P. B., aged 27, of a feeble and unhealthy constitution, was admitted into the hospital on the 28th of September, with all the symptoms of inflammation of the lungs—the breathing 40 times in the minute—sputa frothy, transparent, and very rusty. Dulness on percussion over the fossa infra-spinata. Vesicular respiration superseded by a bronchial blowing;—voice also broncho and œgo-phonic, especially along the base of the scapula. To be largely bled. On the following day slight crepitating râle at the lower angle of the scapula, and this was more distinct on the 30th. The patient was bled on both days. On the 1st October a strong crepitating râle was heard over the supra-spinous fossa, and over the lower third of the infra-spinous fossa. The bronchial respiration and voice still distinct along the base of the scapula; sound on percussion dull; sputa viscid and rusty. Venesection and leeches to be repeated.

2d. The pulmonic symptoms aggravated, and the patient lay on his back in a state of great depression. To be cupped and blistered. 3d. Breathing very laborious, 44, and accompanied with violent action of the nostrils.—Cough frequent; sputa frothy but not rusty. Bronchial respiration below the left clavicle. To be bled to three cups. The clot small, and slightly buffed. He was relieved; and on the 5th, a crepitating râle was heard over all the extent of the supra, and infra spinous fossæ. On the 6th the respiratory murmur began to be blended with the crepitating râle; and this latter sign was appreciable in the left axilla; and the sound on percussion was more sonorous.—From this date the symptoms continued to abate, and the breathing gradually resumed its normal characters.

N.B. The preceding cases are from the Clinique of M. Bouillaud, who is a strenuous advocate of Broussais' doctrines, and is one of the able editors of the—*Journn. Hebdom. No.* 113.

---

### LIX. On the Qualifications of a Physician.

That man is a scientifically informed physician who is well acquainted with, and has, as it were, appropriated to his own use, the results of all the enquiries made at different times by distinguished observers upon the symptoms, course and causes of diseases, and with the precepts of treatment which they have recommended and employed. To become a skilful practitioner, he must understand how to bring this knowledge into operation, and be ready in applying all its rules and deductions to each particular case. This most important talent can only be acquired by extensive researches and by diligent study at the bedsides of patients. Cicero has well said, "Nec medici, nec imperatores, nec oratores, quamvis artis præcepta perceperint, quidquam magnæ laudis dignum sine usu, et exercitatione consequi possunt." The most important attribute in the character of a physician, and indeed of every man who is engaged in the active and practical employment of life, is, after the acquisition of sound theoretical knowledge, the power or faculty of distinctly and correctly perceiving the leading phenomena of the case before him, of tracing the relations of causes and their effects, of reasoning upon them, and of applying the deductions to the remedial treatment. There are many physicians who are excellent theorisers, but who never become skilful practitioners; for with all their accumulated information, they know not how to recognize the individualities of a case, nor to reduce the symptoms to any general rule;—such are all merely book-men, who have acquired no skill in the sick room. On the other hand, there are physicians, and their number is very large, who style themselves sober and useful practitioners, and who treat all cases, after the analogy of previous ones, and the results of their experience in general, and employ, without being able to give any why or wherefore for so doing, certain remedies which they may have used on some former occasion with advantage. Such are the sheer empirics, the routine-men, the despisers of all theory, and the searchers after and triers of every new remedy proposed, by those at least of the same school. Now, although it may appear at first sight to be the easier method of treating diseases upon the analogy of former experience, in reality it is not so; because every new case has something specific and individual in its character; and to arrive at a successful therapeia, the physician ought to attend to the peculiarities which result from the differences of age, sex, constitution, mode of life, employment, and so forth, and to vary and modify his treatment accordingly. This is the business of sound theorizing, and if so, then "to practise without theorising is, in other words, to practise without reflection." There are several reasons which have led many practical men to reject all theory.—With many, the cause exists in themselves;—they are bad reasoners, and in their attempts to discover and to apply the precepts of sound logic to any case, they fall into errors, from the dulness of their perceptions, or from the poverty of their thoughts;—they therefore blame the system; and seldom think of their own incapacities and deficiencies. With others, the distaste arises from their observing the idle dreams and phantasies of enthusiasts, who assume the title of theoretical men, style the vagaries of their brains lofty philosophical speculations, and who have at different times attempted, and not unfrequently too have succeeded, in introducing a system of physic into the schools. But as soon as physicians have learnt to refuse all credence to mere fanciful notions, springing up from darkness, and as soon as they arm themselves with scepticism against such nonsense, by weighing it in the balance of sound reason, and rejecting it as the offspring of an unbridled imagination, then shall the vain strivings of all enthusiasts become more rare and ineffectual, and a wholesome and sound system of theory be no longer despised.

May the hope which Bacon expressed two centuries ago be soon realized—"Speramus et cupimus futurum ut medici nobiliores animos non nihil erigant neque toti sint in curarum sordibus."—*Tiedemann Physiologie des Menschen.*

---

### LX. CASES OF REMARKABLE CURES, BY THE LATE M. DANCE.

CASE 1. *Hypertrophy of the left ventricle, with consecutive engorgement of the liver, ascites, and anasarca.—Antiphlogistic and depleting treatment.*

A. B., aged 18, of a sanguine temperament, was admitted into the Hôtel Dieu under M. Recamier. For 18 months previously he had suffered from violent palpitations, sense of suffocation, which was dreadfully increased by motion, and especially when mounting up stairs. There was general anasarca, except of the face and upper extremities; the face was puffy, the cheeks of a deep red, and the lips of a purplish hue—fluid felt distinctly in the abdomen—liver so much enlarged that it might be traced as far as the umbilicus—dyspnœa, amounting sometimes to orthopnœa—a loud sonorous râle over all the chest; pulse equal and regular, but very small contrasted with the violence of the heart's action, which were so strong as fairly to push off the hand of the auscultator. The patient was bled largely from the arm, and fifteen leeches applied to the precordial region. The leeching was repeated for four days successively; and on the fifth day, a large and deep caustic issue was formed over the region of the heart; it admitted five peas. The patient rapidly improved, and in the course of six weeks was so well, as to be almost able to leave the hospital. The issue was kept open for two or three months longer, and no return of the disease has taken place to the present time; a period of three years.

*Observations.* The preceding case is an excellent example of the good effects of active local depletion and derivation.

CASE 2. *Complete paraplegia, occurring without evident cause, cured by two moxas in the neck.*

A. B., aged 21, of a healthy constitution, had been seized with general weariness and feverish distress fifteen days before his admission. A few pustules had appeared on the body, but these were gone, and now the lower limbs had fallen into a state of perfect paralysis, which had mounted gradually higher, and affected nearly the whole of the body. The palsy had commenced in the right limb, before extending to the left one;—it involved the loss of feeling, as well as of motion;—there was no pain, or stiffness, or any twitching of the muscles. The skin of the abdomen also was completely insensible; the bladder was unable to expel the urine; the bowels were obstinately costive; respiration was performed chiefly by the movements of the diaphragm, the ribs remaining almost motionless; mental faculties and speech unimpaired. Symptoms of paralysis threatened to invade the upper extremities, which were feeble, and could not be easily raised to the head. Pulse small and weak. No cause could be assigned for this malady; the patient had not suffered from any injury or blow, and the spine did not present any irregularity, nor any tenderness when pressed. Sulphureous baths, calomel, and purgative injections were given daily for some time, but with no good; the breathing became more distressed, so as almost to threaten suffocation sometimes; and eschars began to be formed on the parts of the body on which the patient lay. Two moxas were now applied over the spine below the nucha. In eight days the sensibility had returned somewhat, and the incontinence of urine was not so complete. A fortnight afterwards, the palsied parts had recovered their natural sensibility, and began to exhibit some muscular movements; the urine could be retained during the day, and was discharged involuntarily only during sleep. In anotner week, he was able to bend and extend his limbs; but could not rise from bed, nor keep himself erect. The case went on most satisfactorily, and the patient was discharged quite well seven weeks after his admission.

CASE 3. *Chronic inflammation of the neck of the uterus, and tendency to disorganization, cured by mild treatment continued steadily for three years.*

M. S., aged 34, delicate and nervous; mother of five children. Had miscarried some time before, during the sixth month of pregnancy; the placenta had been retained for several hours, and much hæmorrhage had taken place before it came away. Ever since, she had suffered from a constant uneasiness in the hypogastrium, increased by exercise, or before each flow of the catamenia; and she had also a troublesome leucorrhœa. These symptoms continued for a twelvemonth, and then became aggravated; she applied to M. Dance in September 1828; her condition was as follows. A sense of heat, weight and dull pain in the hypogastrium and about the seat, extending to the groins, and much increased on any motion; she was confined almost constantly to bed; when she attempted to walk, she was obliged to bend her body forwards, and advance only step by step; a thick, yellowish, and viscid discharge from the vagina. Her distress was much encreased before each appearance of the catamenia. Examination with the speculum confirmed the presumptive diagnosis of chronic inflammation of the neck of the womb, with numerous tubercular irregularities and tendency to organic changes. The *treatment* consisted in repeated applications of a dozen leeches to the upper and inner parts of the thighs—subsequently two large caustic issues were established in the loins, and these were kept open for the space of three years, during which time the disease existed. Emollient poultices to the hypogastrium, hip-baths and mild injections were also employed. The patient was kept in the horizontal posture for the first year, and quite apart from her husband. In the second and third years she was permitted to go into the country during the fine season, but was enjoined not to omit any part of the remedial measures. Her diet consisted exclusively of milk, farinaceous food, eggs, and vegetables. The symptoms were very little alleviated for the first 12 months, and had not the patient been endowed with astonishing firmness and constant hope, her medical attendants would have left her, with the persuasion that the disease must inevitably become worse and worse. Fortunately they did not; for the second year brought most agreeable changes; by the end of it she was able to rise, walk about, and to enjoy her food. The cure went on progressively, and she was restored to almost perfect health after four years' suffering. The neck of the womb and os tincæ were still, however, caranculated, but not painful on pressure.

The preceding case suggests some very useful and important hints to medical men, that, in certain complaints, they ought not to allow themselves to become impatient from want of success at first; but that, after they have made a correct diagnosis, and laid down a suitable plan of treatment, they should persevere undeviatingly, and without being discouraged.—*Archiv. Gener. Nov.*

---

### III.

### Institute of France.

### I. Academy of Sciences.

*Séance of January* 28*th.*

M. Dutrochet read a most interesting paper, on the "Mechanism of the Respiration of Aquatic Insects." All insects respire by means of numerous tracheæ, which, opening on the surface of the body, admit the atmospheric air; the process is simple and direct in aerial insects, for they, by a muscular action not unlike to that of deglutition, suck it, as it were, into their bodies. Aquatic insects, on the other hand, exercise a double function; sometimes they come to the surface, and then the air is drawn in; but at other times, when they are under the surface, it is subtracted from the water itself, by means of an apparatus which has been called their bronchiæ, although very different from the so-named organs of fishes, for the bronchiæ of insects are for the purpose, not only of admitting the aerated water, but also of separating from it the dissolved air, which then enters the tracheæ, and permeates their bodies.

Now the question which M. Dutrochet undertakes to solve is—"By what mechanism is the separation or evolution of the air from the water effected?" He founds his opinions on the beautiful experiments of Humboldt and Gay Lussac, on the reciprocal transit of certain gases through interposed septa; when a mixture of oxygen, azote, and carbonic acid, in any proportions, is enclosed in a bag having permeable walls, and the bag is then immersed in aerated water, it was discovered by these distinguished philosophers that there is a reciprocal passing through, from within outwards and from without inwards, of the gases; and that this process goes on till pure atmospheric air is left in the cavity. Dutrochet conceived that a similar change might be effected by aquatic insects, which live immersed under water; they are all provided with bronchiæ, which, being very superficial, allow the gases contained within to communicate with the air dissolved in the surrounding medium; the same interchange may, therefore, take place, as we have described above, when a bladder was used as the septum, and probably with much greater rapidity, in consequence of the air in the body of the insect being contained in a number of tubes instead of one bag, and thus a larger surface being exposed to the action of the water. The instinctive motions of the insect also unremittingly accelerate the process, so that the bronchiæ are placed almost, as it were, in a running stream, a condition the most favourable for the conversion of the confined gases into the pure air. The air within the bronchiæ, previous to the change, is probably adulterated or carbonated atmospheric air. Reaumur once observed that a caterpillar (whose organization is fitted only to live in air) continued to live under the surface of water during its larval and crystalis states; it had enveloped itself in its silken shell, and this shell was further protected by some leaves of the "potamegeton lucens." When the shell was broken, it was found to contain air, which, doubtless, had been renewed in the manner we have described above.

*Séance of 4th February.*

Hermaphrodism.

M. Isidore Geoffroy St. Hilaire read a memoir, entitled, " Anatomical and Physiological Researches on Abnormal Hermaphrodism in Man and the Lower Animals." The generative apparatus of either sex may be advantageously divided into three portions or segments, which in many respects are quite independent of each other, and any one of which may present abnormal characters diametrically opposed to, and differing from, the organs with which it is associated. These segments are, 1, the deep-seated organs, viz. the ovaries in the one sex and the testicles in the other, with their separate appendages ; these parts derive their blood from the spermatic vessels. 2. The middle, or intermediate organs, viz. the womb, and the prostate and vesiculæ seminales, with their appendages ; the bloodvessels come from the hypogastric. 3. The external organs, viz. clitoris and vulva, and penis and scrotum; nourished by branches of the external iliac bloodvessels.

The numerous cases of hermaphrodism may be arranged in two classes, according as the hermaphrodism is without or with excess of parts. The first class is subdivided into four groups—1. Masculine hermaphrodism, in which the generative apparatus is essentially male. 2. Feminine hermaphrodism. 3. Neutral hermaphrodism, the apparatus having no determinate sexual characters ; and, 4, Mixed hermaphrodism, the apparatus presenting a real "melange" of both sexes. The second class admits of three subdivisions. 1, Complex masculine; 2, Complex feminine; and, 3, Bisexual hermaphrodism. From the facts adduced in the memoir, the author draws the following conclusions :—

Perfect hermaphrodism, in the anatomical sense of the word, has never been observed. The most complex cases are those in which the deep-seated and intermediate organs are double, the one set being male, the other set female ; but in no case have the external organs been found associated together ; and, in truth, the penis and clitoris could not co-exist, in consequence of their attachments to the pelvic bones, unless, indeed, there was a complete confusion of the ordinary connexions and relations of the parts. Anatomically considered, perfect hermaphrodism is almost impossible, but its physiological existence is indubitable in those animals, as fishes, &c. in which the two halves of the generative apparatus are, in the normal state, much apart from each other, and which animals have no copulation. The frequency of hermaphrodism in general, and of each kind in particular, is very different in the different groups of animals. In the human species, the masculine and feminine sorts are very rare. The rules laid down by medico-legal authors to determine the sex, in many dubious cases, are very unsatisfactory; for, hitherto, only a very small number of the combinations which Nature presents have been distinguished. The difficulty of accurately determining the sex results from the fact, that whereas the internal organs vary so much in number, structure, and arrangement, the external ones preserve their normal number, and the modifications in the other respects, having partly male and partly female characters, are confined within narrow limits. It is, therefore, impossible, that a separate or particular arrangement of the external parts can correspond with each individual or special disposition of the internal organs. M. St. Hilaire remarks, in conclusion, that the legislative division of people into only two classes, viz. males and females, is imperfect; for there can be no doubt that some individuals have really no distinct sex, such as the neutral and some of the mixed hermaphrodites; also those who present the two sexes in nearly the same degree, and are, therefore bisexual.

Adulteration of Salt.

It appears that in many parts of France, this indispensable alimentary substance is frequently adulterated, to evade the heavy tax laid upon it. It is sometimes mixed with saltpetre, Paris-plaster, salt of tartar, Glauber salts, sand, &c. Government has therefore issued a police ordonnance, imposing a heavy penalty on all manufacturers, refiners, and merchants, in whose possession any adulterated salt is found.

*Séance of the 18th.*

LUMINOUS URINE.

A great error has been committed by some, in supposing that the statements of Reiselius and Pettenkover respecting the above appearance, warrant the belief, that the urine is in itself, and while it is discharged, ever phosphorescent; all that these authors have asserted is, that they have observed a luminousness on the ground which has been pissed upon; the correct explanation of which phenomenon is, that certain insects, as the common earthworm, some species of scolopendra, &c. when wetted with urine, give out a degree of light which lasts for about a minute. It is however to be remembered, that phosphorus taken internally causes the stools to be luminous, in consequence of the phosphorus passing through the intestines, and being voided undecomposed.

ANATOMY OF THE MEDUSA MARSUPIALIS.

Dr. Milne Edwards has lately discovered in this zoophyte, (which hitherto has been considered to consist only of a simple mass of gelatine, without any distinction of parts) not only a mouth, provided with tentacula, a stomach, and a number of vessels, but also organs of a more complex structure, analogous to the biliary canals, and to the ovaria of insects.

M. Cuvier had previously established that some of the medusæ are not simple gelatinous masses; for although he could not find any mouth, he discovered numerous suckers, analogous to the roots of plants, and a digestive cavity, under the form of canals permeating every part of the body, and which performed the functions both of a stomach and of a heart. Others of these zoophytes he considered to consist of a homogeneous jelly, and were denominated by him "*agastriques.*"

ON THE ASCENSION OF THE ROOTS OF SOME PLANTS.

M. Dutrochet has observed, that there is a remarkable difference in the cortical substance of those rootlets which mount upwards into the air, from what is found in those which strike into the earth. The genera cactus and pothos furnish the most curious examples of this anomaly; we see considerably sized roots arising from these plants at some distance above the soil, and shooting vertically upwards; and others also, after creeping horizontally along the ground, bending up into the air. In all these ascending roots, M. Dutrochet found that the cortical substance greatly preponderated over the central substance of the roots, and that the cells of the cortical substance decreased in size from within outwards; and not as in ordinary descending roots, from without inwards; in the one set, the largest cells are nearest the central system, in the other they are nearest the bark.

*March 11th.*

Sir Astley Cooper was elected a corresponding member of the Academy, in the section of medicine and surgery, in the room of the late M. Delpech.

Dr. Robert Brown, whom Humboldt has styled "botanicorum facilè princeps," was elected a foreign associate of the Institute. The competitors were Bessel, of Konigsberg—Buck, of Berlin—Dr. Faraday, of London—Sir John Herschel—Jacobi, of Konigsberg—Meckel, of Halle—Mitscherlich, of Berlin—Ærsted, of Copenhagen—and Plana, of Turin. Out of 47 votes, Dr. Brown had 29. The election awaits the royal assent.

Dutrochet, in the name of MM. Serres, Dumeril, and himself, reported upon a manuscript of M. Geoffroy St. Hilaire, the son, entitled "Anatomical and Physiological Researches on Hermaphrodism, in men and animals." He very justly stated, that unless we have accurate and precise notions on the normal formation and development of the various organs of the body, from the earliest period of embryotic life to the time when the fœtus is a perfect being, and fitted for an independent existence, the department of philosophical anatomy which attends to the doctrine of monstrosities, or the science of teratology, as it has been called, can arrive at no satisfactory conclusions on any of the deviations from ordinary structure; hence it is, that, until within a very few years, teratology was only a history or assemblage of disjointed and unintelligible observations, and of crude and whimsical conjectures. Thanks to the distinguished labours of M. Serres, that we now, in some measure, know how Nature proceeds in the simultaneous or successive development of the organs of animals, and have been taught that, in the fœtus of the mammalia, the various organs pass through several transitory conditions or phases of formation, and that these conditions correspond with the normal or permanent conditions of the same organs, in animals which are lower in the scale of existence. Hence we can readily understand, that a monstrosity arises frequently from the progress of these transition-developments being suddenly arrested, and from an organ thereby presenting, at the period of birth, one of its anterior or pre-existent conditions. The discovery of the laws of eccentric development, made by M. Serres, who has shewn that the two longitudinal or symmetrical halves of an animal body are developed, up to a certain point, independent the one of the other, explains very beautifully how the organs of one side may be dissimilar from those of the other side; but it leaves unsolved the curious question of hermaphrodism, or how the organs appertaining to the two sexes may coexist in one of the halves. M. Serres is aware of this, and has most ingeniously attempted to account for it, by referring to the influence exerted upon the development or non-development of organs, by the persistence or obliteration of the bloodvessels destined for their growth. He says that the organs which are nourished by different vascular trunks are independent of each other in respect of their formation, although, perhaps, not of their uses and functions. If we apply this doctrine to the examination of the sexual organs, we at once perceive that the deeply-seated or essential organs of generation, derive their bloodvessels from a different source from what supplies the exterior, or mere auxiliary organs; the one set are supplied by the spermatics and hypogastrics—the other by branches from the external iliac and crural vessels. As we have previovsly explained (*vide* Séance of Feb. 4th), M. Geoffroy St. Hilaire, the father, has, with great sagacity, shewn that the generative organs in either sex are composed of three parts or segments, independent of each other in respect of development, although not of function, and nourished by bloodvessels from distinct sources. This gives us a key to the explanation of many of the cases of hermaphrodism.

Dr. Pamard related a case, to illustrate the good effects of the exhibition of a large quantity of metallic quicksilver, in acting upon and dislodging some balls of lead which had been swallowed, and, becoming obstructed in the intestinal tube, had occasioned alarming symptoms.

Three pounds and a half of the quicksilver were taken on the morning of the 19th Dec. The patient experienced no uneasiness, but what appeared to arise from the great weight of the metal; in the course of three hours he vomited, and severe colicy pains supervened, but none of the mercury was rejected. On the following day the vomiting continued, and, for five successive days, croton oil and oily enemata were given; the stools contained a black powder, and also a quantity of the metallic quicksilver; when it was carefully collected and washed, it was found to amount to 3 lbs. 6 ozs. and 4 drachms; part of it was quite pure—the rest of it contained nine grammes, or about 2½ drachms of lead. Five grammes of the black powder, when analysed, was found to contain five decigrammes, or a tenth of its weight, of lead.

The author concludes his remarks by stating, that the mercury must have acted upon, and amalgamated with, the lead, " since all the morbid symptoms disappeared."

*Séance of the 17th.*

M. Boussingault read a memoir on the thermal waters of the Andes, in South America. He found that the opinion of La Place, respecting the diminution of the temperature of hot springs, in proportion to their elevation above the sea, is correct, except when the waters issue from volcanic rocks; for it appears that, in this case, the local cause which produces the volcanic phenomena has a very marked influence on the temperature of the springs; and M. B. is of opinion that all the equatorial thermal waters, at least those which occur in trachytic beds, derive their heat directly from a volcanic source, as he found that they contained the same gases as are emitted from craters, viz. the carbonic acid and the sulphuretted hydrogen: it is now a question of very curious interest, whether the saline substances also dissolved in such waters are drawn from the bowels of volcanoes. The Memoir concludes with some remarks on the constancy or changeableness of the temperature of the South American springs; the thermometric observations of M. Boussingault sometimes agreed, and at other times differed from, those of Humboldt, made between twenty and thirty years before. Perhaps the earthquake of 1812, which was felt along all the shores of Venezuela, and which destroyed Caraccas and many other towns, may have had considerable influence on the temperature, as well as upon the other qualities, of these mineral waters.

---

### II. Academy of Medicine.

*Séance of 15th January.*

M. Maingault made some remarks on tracheotomy, and on the introduction of caustic substances into the trachea, in croup and diptherite. He recommends that the tube be opened very cautiously, and that the opening be made small at first, and gradually enlarged, to prevent the too rapid entrance of the air, which might cause asphyxia. The use of caustics he condemns. M. Velpeau, on the other hand, denies that any danger has ever followed a free opening into the trachea, and ridicules any apprehensions on the score of asphyxia. As to the employment of local caustics, M. Velpeau adverts to four cases of success, treated by M. Bretonneau, and one by M. Trousseau. A solution of nitras argenti was introduced into the wound by means of a small bit of sponge wet with it, and a few drops permitted to flow into the trachea. There can be no doubt of the efficacy of the topical use of the caustic in " angine couenneuse," or diptherite.

### Double, or Bilobed Uterus.

M. Moreau presented a very beautiful specimen of a uterus divided completely into two equal lateral halves; each provided with a tube and an ovary; each separated from the other by a double partition, and each having distinct necks and mouths into a single vagina. The woman died after an accouchement;—the fœtus was a male, and had been developed in the left cavity; and therefore not in accordance with the whimsical idea that the right ovary furnishes the male, and the left one the female germs.

### Disease of the Kidneys.

M. Tanchore presented, for the inspection of the Academy, a diseased kidney, which had acquired the size of a child's head, and caused during life such a prominence of the abdomen, that some physicians had supposed that the liver was much enlarged, and was the seat of the disease. The patient had suffered very little pain, although his urine was almost always purulent. A sinapism had been very imprudently applied to one of his limbs, which had become œdematous;—gangrene was the consequence, and of this he died. On dissection, the iliac and femoral arteries were found obstructed with masses of lymph. The left kidney was enormously enlarged, and embossed, or lobulated on the surface; each lobule containing a deposit of pus. A large calculus was imbedded in the centre of the organ, and surrounded with a cyst of lardaceous matter.

Bilobed Urinary Bladder.

M. Velpeau exhibited a specimen of this rare formation; the second, or subsiding pouch, was small and situated posteriorly, and communicated with the anterior pouch by an opening near to the trigone. A calculus was found in the anterior pouch;—it could have been extracted only by the high operation, as the incision in the lateral mode must have been made into the posterior bag.

*Séance of* 22*d*.

M. Dubois read an interesting paper on the probable causes of the general presentation of the head in labour. The old opinion that it depends on the greater weight of the head, which thus gravitates to the depending part, will not well stand the test of enquiry. M. D. found that in numerous experiments, wherein he placed the body of a fœtus on the surface of water in a bath, and allowed it to subside; the back and shoulders, and not the head, reached the bottom first.

Moreover in women, who keep the horizontal position during the whole, or greater part of gestation, the presentation is still usually that of the head; and even with acephalic monsters, the same very generally holds true. It is well known that the head of the fœtus is proportionably larger and heavier in the early, than in the later months of pregnancy;—now a necessary consequence of this should be, according to the old interpretation, that the head be invariably dependent; but is it so? No, certainly; for the earlier the miscarriage, the more frequent is an abnormal presentation. The following table shews this very satisfactorily.

| Abort. before 7 mo. | Head. | Pelvis. | Shoulder. |
|---|---|---|---|
| 1829.... | 30 .. | 22 .. | 7 .. 1 |
| 1830.... | 35 .. | 16 .. | 18 .. 1 |
| 1831.... | 23 .. | 13 .. | 9 .. 1 |
| 1832.... | 34 .. | 14 .. | 17 .. 2 |
| | 122 | 65 | 51 | 5 |

According to this table, the number of the pelvic presentations is to that of the cephalic as 4 is to 5; whereas, at the full period of gestation, it is as 1 to 20. Again, in the lower animals, whose pelves are so inclined as to preclude the operation of gravity, the fœtus' head is towards the os tincæ.

M. Dubois having thus objected to the former hypothesis, advances one of his own, and explains the cause of the head presenting "to a desire, or besoin d'etre, which Nature has impressed upon the fœtus," as if the little one was instinctively, or even voluntarily led to push out its head foremost!! [Just as we see people prefer a station near a door or window in a very crowded room —*Ed.*] In proof of the opinion that the fœtus is endowed with "déterminations instinctives, ou voluntaires," M. Dubois adverts to the greater frequency of abnormal presentations, when the fœtus has died; to its movements while in the womb, induced by firm pressure on the abdomen, change of position of the mother and so forth; to its struggles when the umbilical cord is compressed, so that it has been known that the liquor amnii has rushed into the thorax dilated by the violent effort of the muscles of inspiration; and lastly, to the admitted act of instinct in the case of the chick, when it breaks the shell of the egg to make its escape. M. M. Virey, Velpeau, and Capuron objected to the views and reasonings of M. Dubois. The memoir was however much applauded by the Academy, and ordered to be printed in their collection.

Mucous Fever.

A report was read, of an epidemic which has lately prevailed at Besancon and the neighbourhood. This fever is the pituitous fever of the ancients, and a gastro-enterite, as a matter of course, of the Broussaian or physiological school; and correctly too, for there is certainly an inflammatory affection of the mucous lining of the alimentary canal, even although the symptoms do not very strongly indicate the mischief. The observations of M. Andral are so pertinent, that we cannot do better than insert them.

"In 98 out of 100 cases of essential fevers, some morbid changes are found upon dissection; but we are not to expect any well-marked or determinate relation between these changes and the preceding symptoms during the lives of our patients. If we were to judge by post-mortem examinations only, we should often believe that inflammatory, bilious, catarrhal or mucous, typhoid or adynamic, nervous or ataxic fevers, were one and the same disease. External symptoms, however, sufficiently attest important differences between these, and these differences require corresponding differences of the therapeutic indications." Having proved, by most extensive researches, that the appearances of the tongue do not faithfully indicate the state of the intestinal canal, M. Andral inculcates the necessity of diligently attending to the signs furnished by it, for these signs he deems to be more useful in directing us to a proper treatment than any "*cadaveric lesions.*" A red tongue, says he, although it does not necessarily announce intestinal irritation, indicates the propriety of antiphlogistic remedies—an enlarged, white, or yellow tongue requires emetics—and a dry, black tongue forbids, whatever may be the state of the digestive organs, the use of whatever enfeebles or exhausts the system.

[Although somewhat out of place, we are tempted to introduce some remarks by Professor Lobstein, of Strasburg, on the subject of fevers, as they much tend to confirm the opinions of the distinguished French pathologist.]

I am satisfied, says he, that the post-mortem appearances, in typhus fever, are not to be considered as the actual exciting causes of the disease, and of its symptoms; for the serous infiltration of the membranes of the brain is very frequently found in cases which have no relation with nervous fever. The injection or inflammation of the gastro-intestinal mucous membrane is by no means a constant phenomenon, as we have been assured repeatedly by our own researches, and as is testified by the observations of MM, Petit, Louis, Martinet, and Andral; and, lastly, the dothinenteritis, or ulcerative inflammation of the mucous glands of the bowels, so much insisted upon by Bretonneau, although very frequently met with, is sometimes altogether wanting, as Alison, of Edinburgh, and Neumann, of Berlin, have proved; and, moreover, it occurs in other diseases, as in scarlatina, phthisis, &c. without producing any of the symptoms of typhus fever. We are, therefore forced to reject all these theories, if they are propounded as exclusive; sometimes they, or at least the facts on which they are said to be built, are right, and sometimes wrong; and, in very numerous cases of fever, we can recognize only an affection of the nervous centres as the exciting cause of the diseases.—*Vide Arch. Gener. Janvier.*

*Séance of 26th.*

A very singular case of paraplegia, accompanied with complete constipation, and the suppression of urine during the space of 14 years, was communicated by Monte-Santo, of Padua.

Several years before the accession of the paralytic symptoms, the patient had sustained a fall from a height upon his back; none of the vertebræ were fractured, but the concussion had been so severe, that he never recovered entirely from its effects. For 14 years his appetite was strong, and he eat his food heartily; after each meal, he drank largely of water, and after the lapse of two hours, he felt obliged to take another large draught, in order to induce the vomiting of such food as he had swallowed; this painful operation recurred every day, from two to five hours after eating. About once a month, he had the sensation of, as it were, a ball, which rose from the stomach to the mouth; this lasted for four hours or so, and then a large quantity of feculent matter, mixed with blood, was vomited. On no occasion was there the least appearance, not even any smell, of urine in these egesta; the renal secretion seemed to be quite suppressed. Since March, 1829, the fæcal vomitings have never occurred; but each repast is rejected more quickly after it has been swallowed than before. The digestion and assimilation of the food still, however, go on perfectly well, for the patient has of late become so plethoric, that he has repeatedly required bleeding. He still lives, and MM. Graefe and Frank have visited the individual, and confirm the accuracy of the above statements.

A similar case was communicated to the Academy, in 1823, by M. Denis. There was no alvine evacuation per anum, nor excretion of urine, for 72 years. Probably the kidneys had become atrophied.

*Séance 5th March.*

### Singular Variety of Hermaphrodism.

A person was admitted on the 7th April of last year, as a male patient into the hospital La Pitié, labouring under cholera. He died next day; he gave his name as Valmont, said that he was 62 years of age, was a widower; and that his trade was that of a hatter. His aspect did not lead to any suspicion; and he had a beard "assez epaisse."

M. Bouillaud was therefore not a little surprised upon finding a distinct and well-formed uterus, when the body was opened; his constant engagements at that busy period prevented him from prosecuting minutely the examination, and he therefore removed all the organs of generation and put them into spirit. M. Manec, the eminent anatomist, carefully dissected them. The penis was of ordinary size and well formed, having a glans and prepuce; the meatus urinarius was not exactly in the centre of the glans, but somewhat nearer its lower part; the scrotum was rather small, but otherwise quite masculine, being brown and puckered; divided by a longitudinal raphe, and shaded with hair; there was however no trace of testicles; nothing but such cellular tissue as we find in the labia pudendi, or nymphæ. The mons veneris was fuller and more prominent than in man; the internal organs were two ovaries, two fallopian tubes, and a well-formed uterus, of the usual size in the virgin state; it was altogether "d'une conformation, qui ne laisse rien à désirer," and situated between the rectum and bladder; it opened by a regular os tincæ into a vagina, which was of an average size, and two inches in length; where the vagina was close to the neck of the urinary bladder, it suddenly contracted, and at the membranous portion of the urethra it was reduced to a very small tube, which after turning upwards opened by an orifice of the diameter of about two millimetres, into the membranous portion, so that the urethra was in fact the continuation of the vagina. Beyond the opening, the urethra had all the characters of the male one; at the neck of the bladder, it was surrounded by a prostate; and a distinct verumontanum, with the orifices of the prostatic ducts opening at its side, was found. The corpus spongiosum urethræ, and the two corpora cavernosa penis were of the usual size and appearance; the acceleratores urinæ were very large; Cowper's glands also existed. There was no trace of testicles, vasa deferentia, or vesiculæ seminales; a sort of round ligament passed through the inguinal canal.

It is stated that the general form and configuration of the body was intermediate between that of the male and of the female sexes; and M. Bouillaud is of opinion that the person was in the "juste milieu" between a man and a woman; he indulges in a long train of amusing imaginings on the character, actions, and general conduct of the individual; in allusion to his marriage he rapturously exclaims "Comment s'est il comporté dans l'act du coit? Quels transports pouvait-il éprouver aupres d'une femme?" and wishes the Academy to solve the question, whether Valmont "a veritablement ressenti l'aiguillon de la chair; et dans ce cas si c'est plus specialement l'aiguillon de la chair masculine, ou celui de la chair feminine; ou bien encore s'il aura été tour-a-tour en proie au stimulus de ce double aiguillon; ou si par une sorte de neutralisation d'une sexe par l'autre, Valmont sera resté dans un état d'indifference en matiere generatrice." His less imaginative bretheren MM. Geoffroy St. Hilaire and Manec, content themselves by shewing that even the above is not an instance of true hermaphrodism; we must distinguish the organs of reproduction, and those of mere copulation; there may be an amalgamation, or co-existence of the latter, but not of the former.

Those who wish minuter details must consult the March No. of the *Journal Hebdomadaire*.

*Séance of March 26th.*

M. Girardin made a report on the state of vaccination in France, during the year 1831. It appears from the documents presented by him, that the number of persons vaccinated has diminished progressively since 1827.

---

*Spirit of the Foreign Periodicals.*
*(Continued.)*

LXI. Satyriasis, following a Blow upon the Occiput.

A man, aged 53, of very quiet and religious habits, accidentally struck the back part of his head and neck against one of the corners of his bed. There was considerable contusion, but no farther injury. Very soon afterwards he became quite satyriacal; formerly modest and decorous, he was now so vehemently salacious, that no woman could approach him without risk of being insulted. Even his own daughters were not safe from the rage of his lust. For three months, this state of erotic excitement lasted; his physical and intellectual powers became enfeebled and childish, so overpowering was the predominance of this one passion. After a fit of furious anger at his wife, for refusing him conjugal rights, he fell into a convulsion, complained of an intense pain at the crown of the head, and, from that moment, all uneasiness at the occipital region ceased—the satyriasis left him, and in its place a religious delirium, indicated by a constant muttering of prayers, succeeded. He became slightly paralytic on the left side, and died on the 8th day after the sudden change in the symptoms. Unfortunately, permission could not be obtained to examine the encephalon after death; and we are, therefore, left in the dark as to any organic changes.

The metastasis of diseased action from the cerebellum to the vertex (where the organs of veneration and hope are situated), and the corresponding changes in the dominant emotions, are curious and worthy of notice. Dr. Gall has stated, "that wounds and blows on the occiput or nucha have been followed by inflammations of the genital organs;" and M. Voisin, in his work on the Moral and Physical Causes of Mental Diseases, has the following pertinent remark—"the material condition of satyriasis resides in the encephalon; and, in all cases, the inordinate passion is in proportion, either to the original over-activity of the cerebellum, or to the occasional circumstances which have caused the organ to be violently excited." In the treatment, therefore, of such cases, our attention ought to be directed specially to the remote or essential cause. M. Voisin has adduced numerous cases, in confirmation of the superior efficacy of such a ratio medendi. Satyriasis and its consequence, masturbation, are not unfrequently met with in such hydrocephalic patients as have arrived at puberty; and Dr. Gall has insisted upon this, as a strong argument in favour of the encephalic origin of the disease. Those who have had an opportunity of examining satyriacal patients will agree with us, when we state that the orgasm of venery frequently does not pass away with the seminal emission, but that the penis will remain erect, and the semen be every now and then squirted out, for a considerable time. The intellectual powers are almost always greatly impaired, the miserable wretches living under the tyranny of their lusts. Now it is not easy to render a philosophical interpretation of these facts, except upon phrenological principles; and if they be admitted as true, the interpretation is at once obvious and satisfactory. There is an every-day occurrence which deserves notice, as it is in the power of any one to test its reality on himself; we allude to the venereal desire being very generally excited by lying flat upon our back. It seems as if the cerebellum was stimulated by the slight congestion thereby induced.—*Transact. Medicales.*

---

### LXII. Anomaly of the Arch, or Curve of the Aorta.

Dr. Vidal examined the body of an old woman, in whom he found that the arch of the aorta was bent forwards, or sternad, from left to right; it then descended in front of the right pulmonary vessels, passed behind, or dorsad, to the pericardium, and recovered its normal situation before piercing the diaphragm. The vessels given off from the arch were, first, the left carotid, then the right carotid, the right subclavian, and, lastly, the left subclavian, which, before reaching the clavicle, passed between the œsophagus and spinal column. The remains of the ductus arteriosus shewed that the canal had, originally, existed between the left pulmonary artery and the left subclavian.—*Journ. Hebdom.*

---

### LXIII. Two Cases of Œsophagotomy.

A soldier, while eating soup, accidentally swallowed a bone. He applied immediately for assistance to the surgeon of the regiment, and a variety of means was tried to remove it; but all in vain. On the fourth day after the accident, he was put under the care of M. Begin. By introducing a caoutchouc sound down the pharynx, he found that it passed about seven or eight inches, and was then obstructed by a foreign body, which was hard, and immoveably fixed in its place; this seemed to be in the œsophagus, immediately below the level of the cricoid cartilage. No forceps could reach this depth, and the severe pain caused by the firm pressure of a probang, forbad any attempt to push the bone down into the stomach. The patient experienced much anxiety and distress in his breathing. It was proposed to give him an emetic, but the reporter states, that " la deglutition etoit trop difficile, et ce plan ne put recevoir d'execution" (the wherefore is not very obvious.) On the sixth day, the saliva was mixed with purulent matter; but the bone stuck as fast as ever, and now three grains of tartrate of antimony were exhibited; the patient vomited freely, and felt himself relieved; the probe did not, however, indicate any favorable change in the position of the bone. The respiration was moderately easy, and he could swallow fluids with little inconvenience. The quantity of pus which escaped from the mouth had increased in quantity for some days past.

As all means to dislodge the bone had been tried without effect, M. Begin resolved on the 12th day after the accident, to attempt its extraction, by cutting down upon the œsophagus. An incision was made between the trachea and the inner margin of the sterno-mastoid muscle, extending from the sternum, to the upper edge of the thyroid cartilage. The omohyoideus muscle was divided across, the trachea drawn to the right side of the wound, and the carotid artery, jugular vein, and par vagum, to the left; a branch of the superior thyroid required the ligature. On carefully continuing the dissection, a drop of pus followed the stroke of the scalpel; a director was then carried deep into a large sac, lying close to the gullet, and a bistoury carried upwards and downwards, so that the anterior parietes of the sac were divided to nearly the extent of the outward wound; a large quantity of pus mixed with shreds of cellular substance flowed out; the left fore-finger was now introduced, and the thin intervening septum of the walls of the œsophagus was distinctly felt; by gently rubbing and pressing this, it gave way, and the finger passed into the gullet itself; still no foreign body could be found; a mouthful of drink was given, and this escaped from the wound. The opening in the œsophagus was cautiously enlarged by the bistoury; a large vessel, probably the superior thyroid, sprung; the bleeding orifice could not be found, but was at length secured by passing a needle fairly round and thus inclosing a button of the surrounding parts in the ligature; the finger being now passed as deep as it could be carried, felt the bone, impacted in the tube of the œsophagus, nearly opposite to the upper piece of the sternum; a bent forceps was then introduced, and attempts were made to lay hold of the bone; but in every attempt, it seemed that the walls of the œsophagus got included between the blades. A blunt-pointed tenaculum could be used more easily, by inserting it round the lower surface of the bone, and then gently dragging it upwards, till at length it was fairly extracted; a quantity of pus and of sloughy cellular substance escaped at the same time. The operation occupied 25 minutes. Absolute abstinence was enjoined, and the mouth was only moistened occasionally, to relieve the thirst; on the following day, an elastic gum tube was passed into the stomach, and some broth given. The progress of the case was altogether most favourable; the wound gradually healed, the food ceased to escape by the orifice, and the patient ultimately quite recovered.

*Case* 2. B. J. was brought to the hospital on the 30th Feb. 1831. On the preceding day he had swallowed a bone, which stuck in the gullet, and could not be removed. An emetic had been given, and although free vomiting was induced, no benefit followed. So firmly was it impacted, that no tugging with forceps, or with probangs passed beyond it, was of any avail. It did not altogether block up the tube, for small brobangs, or rather probes curved at the end, might be introduced further down than it lay, although with some difficulty; considerable purchase might therefore be employed, during the withdrawal of these; but in spite of every effort, the foreign body could not be dislodged. The respiration and deglution being easy, the surgeons were unwilling to resort to an operation at present, as there were good grounds for expecting that the bone might be gradually loosened, if not by the motions of the canal, at least by suppuration. But these hopes were speedily found to be fallacious, and the patient suffered such distress that he was urgent that something be done for his relief. On probing the œsophagus, it was thought, that the bone had descended somewhat as it could not be seized so easily as hitherto with the forceps. M. Begin therefore resolved to operate, and the operation was performed on the eighth day after the accident. The steps of it were very similar to those in the preceding case; the integuments were freely divided, the trachea and œsophagus separated from the sheath of the great cervical vessels, and then the finger introduced deep into the wound; the bone was felt through the walls of the gullet, somewhat below the level of the upper edge of the sternum. The bistoury was carried along the finger as a probe, in order to avoid any injury to the inferior thyroid vessels, and a small opening was made into the œsophagus; this was gradually enlarged, till the finger could enter freely into the tube; the bone being now felt distinctly, a pair of long and curved forceps was introduced, and with much difficulty the bone extracted. Not above two table spoonsful of blood were lost.

The subsequent treatment was the same as had been so successfully adopted on the former occasion; all the food was introduced by means of a stomach tube. On the 4th day, a healthy suppuration was established, and the wound slowly closed. He was discharged in six weeks perfectly well.—*Journal Hebdom.*

The boldness and dexterity displayed by M. Begin in undertaking and successfully performing the hazardous operation of œsophagotomy, shew that he is one of the best surgeons in the French metropolis. His example has been ably followed by Mr. Arnott, in a case which occurred lately at the Middlesex Hospital.—Ed.

### LXIV. Extirpation of a Tumour from the Neck. Death from the Admission of Air into a Vein.

A young girl was admitted into the La Charité for the purpose of having a large tumour, situated on the side of the neck, removed. M. Roux was the operator. The dissection was tedious and difficult; and when nearly completed suddenly a peculiar whizzing sound was heard, as of air rushing through a small aperture into a vacuum—the patient uttered a feeble cry, and became convulsed; the respirations were deep and only at long intervals, a mucous râle could be heard distinctly in the chest, the pulse was hurried and very weak, and speedily all the appearances of death ensued. Frictions on the epigastrium, tickling the nostrils and dashing cold water on the face, gradually resuscitated the patient; the speech at first was confused and stammering, and a quantity of mucus and saliva dribbled from the mouth. The operation was completed by passing a ligature round the base of the tumour. For the five following days no bad symptoms appeared; on the sixth, the mass of the tumour was in a state of sloughing and emitted an abominable stench,—it was therefore removed. On the next morning the patient became comatose and died.

*Dissection.* By carefully examining the situation of the tumour, it was ascertained that the sheath containing the carotid artery, jugular vein and par vagum, had been opened during the operation, and that the vein had been divided across; the lower or sternal end lay gaping; and on introducing a probe it passed readily down to the junction with the subclavian. The bronchial tubes were filled with a frothy mucus, and there were several emphysematous points under the pulmonary pleura. The cavities of the heart were found quite empty; when the aorta was pricked at different parts of its course, bubbles of air mixed with a bloody serum oozed out; even the same was observed in the common iliacs, although in a less degree. Nothing abnormal was found in any of the veins; and no air could be discovered in any of the cerebral vessels.

*Remarks.* Bichat was of opinion that in such cases as the preceding, the death is attributable to the paralysing effects of the contact of air on the brain. Nysten and Magendie have ascribed it to the forcible and permanent dilatation of the heart. M. Leroy d'Etiolles, in his memoir on asphyxia, endeavours to prove that it may be occasioned in three different ways, either by the deleterious agency of the air on the cerebral substance; or by the pulmonary emphysema; or lastly by the disturbance of the actions of the heart. The extreme rapidity of the death makes it probable, that the heart is the organ primarily affected; in some cases the patient has died in a moment; the centre of the circulation suddenly ceasing to act.—*Journ. Hebdomadaire.*

---

### LXV. Insalubrity of Paris.

A commission of health was lately appointed to inquire into the causes of the great unhealthiness of the quarter of St. Martin in Paris. Its situation is low-lying, and a great part is not above the level of the Seine; the soil on which it is built is therefore alluvial and excessively damp; there are moreover some manufactories, such as those of "poudrette" (human dung reduced to powder as a manure) which must inevitably be injurious to the residents; there is the canal of Saint Martin, constantly giving out its malarious miasmata; there is the densely crowded state of the buildings, the huddled together population of the lowest classes of society, living in rooms or hovels where they have scarcely air to breathe; besides, this air is infected with the most noxious and abominable effluvia from the disgraceful condition of most of the privies and necessaries, private as well as public; they have been so constructed as to permit the escape of the urine and much of the fluid filth, (which thus runs down in the kennels of the streets) in order to save the expense of frequent cleanings; and many are situated close to, and alongside of the wells, which afford the water that is drunk by the inhabitants: various plans have been suggested with the view of obviating this disgusting state of things. In our opinion by far the best would be, a police order to shut up all deep privies or "fosses," and to substitute in their place "sieges," fitted with removeable reservoirs, on the plan of the portable water-closets, or of Chaumette's apparatus. "Some have asked, why is Paris not provided with drains and sewers, as London is, to carry off into the Seine, as into the Thames, all the "immondices?" but it seems to have been forgotten, that the topographical position of the one city is very different from that of the other, and that the Seine cannot be properly compared with the Thames; and even supposing that sewers might be conveniently made, we deem them unnecessary in many respects; for surely the "immondices" of a city were never destined to infect and pollute the waters of a river; but they should rather be used to enrich and fertilize the earth; a system of sewers may therefore be fairly objected to, as injurious not less to the hygienic than to the agricultural interests"!—*Revue Med.*

---

### LXVI. Imperfect Interauricular Septum, without Cyanosis.

A child aged seven years died of small-pox. On dissection it was found that the septum between the two auricles of the heart was reticulated and made up of an open mesh of fibres, between which any fluid might pass freely from one side to the other. The child had not during life exhibited any symptom of cardiac, or pulmonic distress; there had been no lividity of the surface, no dyspnœa, and no chilliness of the skin; and indeed, had the body not been examined, the imperfection would never have been suspected; the heart was hypertrophied, and there was a considerable quantity of serum in the pericardium. M. Pigeaux states that out of 13 cases of open, or of imperfect interauricular septum, which he has met with, only two were accompanied with cyanosis.—*Revue Medicale.*

---

### LXVII. Extirpation of a Diseased Ovary.

A. D. aged 31, mother of four children; during her last, the fifth, gestation, the abdomen was so enormously distended that it was thought that she was pregnant of twins. The size, however, was not much abated after delivery, and it was then ascertained that it was caused by an enlarged ovary on the right side. A trocar was introduced, and 14lbs. of a serosity were drawn off; the limits of the swelling could now be more easily defined; and, as it felt hard and fluctuating, the trocar was inserted at another point, and 12lbs. more of a watery fluid were evacuated. Dr. Ehrhartstein decided on its extirpation, which was performed eighteen weeks after delivery.

On exposing freely the diseased mass it was found to have contracted adhesions to the surrounding parts; these were cautiously severed, and the tumour extracted in fifteen minutes from the commencement of the operation. Only three vessels required ligatures. Severe febrile symptoms supervened, and lasted for several days. On the eighth day a profuse discharge of bloody serum and of gas escaped from the wound, with great relief to the patient. In nine weeks the wound had healed completely.

The diseased ovary weighed 12lbs. and when cut through exhibited numerous cavities, partly filled with a serosity. The three vessels which had been divided, were of the size of writing quills.—*Med. Jahr. des Oester Staats.*

---

### LXVIII. Example of the Purulent Diathesis, with an Eruption of Ecthyma Cachecticum.

A man aged 40, was admitted into the wards of the La Charité Hospital, under the care of M. Rayer, for what was considered a slight rheumatic affection.

By his own account, he had been ill only for a few days, and all that he complained of was flying pains in the right arm, especially about the elbow. His case attracted little notice, till an abscess formed immediately above the joint; there was little or no pain, but only a redness and fulness of the part, which became gradually softer and softer to the touch. At the same time the constitutional symptoms experienced a most unfavourable change; extreme prostration, sunk features, weak tremulous pulse came on; and in two or three days the man was in the last stage of a most malignant typhoid collapse. An eruption of livid papules, or hardened bases had appeared on different parts of the surface; and here and there were puffy swellings, which had proceeded to suppuration. On the neck and upper part of the chest, there were numerous large pimples, or little tumors of different sizes, from that of a pea to that of a nut, all tending slowly to the formation of pus at their summits. The patient died in a state of muttering delirium.

*Dissection.* In various parts of the body, especially in the substance of the muscles, abscesses were found; the largest of these were about the size of a pigeon's egg. In other parts they were more superficial, being situated immediately under the integuments. On cutting through the muscles of the right leg, an immense purulent deposit was discovered; the tibialis anticus was reduced to shreds and the bone laid bare. A similar abscess, but not so extensive, existed in the corresponding part of the left leg, and in addition to numerous superficial abscesses, several were found imbedded in the tissue of the gastrocnemii. The shoulder, elbow, wrist, hip, knee, and ankle-joints were all affected; the synovia had become thick, turbid, and of a greenish-yellow colour, and was much increased in quantity. No traces of phlebitis were discovered anywhere. One small abscess was found in the right lung; the heart healthy and likewise the abdominal viscera, with the exception of the spleen, which was much enlarged, and so softened as to be easily lacerable.

*Observations.* The preceding case bears considerable analogy to the worst examples of phlebitis, and the late M. Dance, in an excellent memoir published in the 18th vol. of the Archives Générales, has the merit of first directing our attention to the resemblance. In a patient in whom numerous abscesses had formed in different parts of the body, and a cachectic ecthymatous eruption also had existed, he found upon dissection abscesses in the muscles of the extremities, vomicæ in the lungs, and the left jugular, portal, and hepatic veins much inflamed. Whether the purulent deposits are dependent upon or are merely co-existent with the phlebitis in such cases, is a question of much pathological interest, and awaits future researches for its solution. The case which we have detailed is indeed hostile to the hypothesis; for not only the veins in the immediate neighbourhood of the abscesses, but also the splanchnic veins were most carefully examined, but no trace of phlebitis was to be found.—*Archives Générales.*

---

### LXIX. Case, in which several Biliary Calculi were discharged outwardly from an Abscess.

A man, aged 48, applied at the La Charité for advice, respecting a swelling which made its appearance several months before, at the lower edge of the false ribs on the right side. It was accompanied with constant severe pain; but there was neither vomiting, nor any symptom of jaundice; diarrhœa had occurred at intervals. The swelling, at first very painful and hard, became gradually softer; an eschar was formed by rubbing caustic potass on its surface; and when this separated, a considerable quantity of reddish purulent matter escaped. The pains however did not abate. This state of things continued for upwards of five months, the purulent discharge going on all this time, when the patient felt as if some rough or pointed body was irritating the wound in the side. One of his companions drew it away by means of scissars; and after its removal, a copious flow of pus followed, with great relief to the pain and general distress. On the recurrence of these, he applied at La Charité, and now it was ascertained that the substance which had been withdrawn, was a biliary calculus; it was of the size of a pea. Upon probing the wound, the point of the stylet came in contact with something hard, rough, and moveable; when extracted, it proved to be another biliary calculus. Fortunately the constitutional disturbance was not great; there was considerable emaciation, but the appetite was good, the bowels regular and healthy, and the pus from the wound laudable.

During the subsequent week, several calculi was discharged, and the patient improved in every respect. Cases similar to the one now reported have been recorded by various authors, as Petit, Sœmmering, Cheselden, &c. &c. Those who are interested to know the particulars are referred to the paper in the March number of the *Archiv. Générales.*

---

### LXX. Treatment of Hooping Cough and Measles.

A very fatal epidemic of these diseases lately prevailed at the same time in Bischwiller and the environs. A vast number of children died. M. Luroth, having been foiled with the treatment ordinarily used, had recourse to frictions with strong tartar-emetic ointment rubbed on the chest and epigastrium; the result was most gratifying, and could not possibly be mistaken. The ointment consisted of a drachm and a half, and sometimes two drachms and a half of the salt mixed with an ounce of lard; the strength of it was proportioned to the age of the young patients; the strongest ointment was applied to all children above two years of age; half a drachm was rubbed in twice a day, till a copious eruption of large painful pustules was brought out, and the eruption was kept up for a few days by an occasional application. In thirty-eight cases occurring in children from the age of one to fourteen years, thirty-four were speedily cured by antimonial frictions, combined with emetics and emollients. The space of time varied from six to twenty-four days;—the average was twelve days. The failure which has not unfrequently attended the employment of this ointment in the hands of others, the author attributes to the insufficiency of its strength, and to want of perseverance in its use, without considering it an infallible or specific remedy against hooping cough, and the pulmonary complications which so often accompany measles, he regards it at least as by far the most certain means of cure. The sympathetic eruption on the organs of generation was observed several times. —*Gazette Medicale.*

---

### LXXI. Treatment of a Goitre by Seton.

In a late case at the Hôtel Dieu, Dupuytren established a free suppuration by means of a seton over the tumor. In three weeks the size of it was reduced by two-thirds, and a complete cure was speedily anticipated.—*Lancette Française.*

---

### LXXII. Lectures on the Cholera, delivered at the College of France. By Professor Majendie. Octavo, Paris.

The following analysis of the leading phenomena observed in the cold or blue stage of cholera is given by this able physiologist and physician, as leading us to what he considers to be the correct view of the cause or rationale of the disease. First of all occurs the suspension of the circulation; then come the cramps and convulsions, and, lastly, the profuse vomiting and diarrhœa:—Such are the essential and pathognomonic symptoms of the disease; and, by attending to the succession of these, we may explain all the other "epiphenomena," or accessory symptoms. Well, the first link of the chain, our author tells us, is the stoppage of the circulation; this is the fundamental doctrine of his theory—the starting point for all future enquiries. The cause of this stoppage, he says, is a weakening or paralysis of the ventricles of the heart; the piston ceases to act, and the blood stands still. That the blue colour of the skin is owing to this "stasis" of the blood, is incontrovertibly proved by the results of experiments, in which the heart's actions can be arrested, and we may then, at will, increase or diminish the cyanosis, by permitting or interrupting the communication of the heart's impulse with the remote venous ramifications. We need no other argument, to be satisfied that the blue stage of cholera is to be attributed to a simple stasis of the blood, and has nothing to do with inflammation.

But the mere arrest of the circulation will not explain some of the other phenomena of this most anomalous and puzzling disease; for upon what principle are we to account for the muscles retaining their contractility, the glands, as the mammæ, for example, continuing to secrete, and the mind preserving its powers unaffected? M. Majendie is fully aware of the difficulty of solving the problem, and candidly confesses his inability to do so. By careful auscultatory examinations, he has satisfied himself that the severity of the symptoms is always proportionate to the feebleness of the heart's actions, and that these become actually extinct in the very worst cases. The rice-water evacuations are directly derived, he says, from the mesenteric arteries and veins, and, in confirmation of this opinion, he has adduced some experiments, in which he forcibly injected watery fluids into these vessels, and found the intestines filled with a discharge, very like to what we observe in fatal cases of the disease; these evacuations are, therefore, to be regarded as consisting of the serous portion of the blood, blended with the mucous secretions. Every thing demonstrates that they are not the product of irritative or of inflammatory action. Having satisfied himself that the blood in cholera undergoes serious changes, that it loses a large portion of its serum and of its saline constituents, and becomes a viscid homogeneous, syrup-like fluid, M. Majendie was induced to try the effect of injecting into the veins an artificial serum, warmed to the heat of the body; in only one case, however, was this method successful in restoring the patient from the collapse, and the restoration, unfortunately, was only temporary, for in a few hours she again sunk, and died. In short, we must not allow ourselves to believe that it is merely the want of the serum which characterizes cholera blood; other changes have taken place in it, some appreciable, and others not so, and, till we have been taught a more perfect animal chemistry, we fear that any hopes of curing the disease by injections into the veins are fond and fallacious. The author was anxious to ascertain the effects of cholera blood, introduced into the circulation of a healthy animal: small quantities, he found, did not create much disturbance; but, when the quantity amounted to eight ounces for a moderate-sized dog, the animal speedily died, with symptoms much resembling those of cholera. It is unnecessary to say any thing of Majendie's treatment—it is well known, and is quite as inefficacious as any other.

---

### LXXIII. Academy of Medicine.
*(Continued.)*

### *Séance of April 16th.*

#### Diptherite—Tracheotomy.

M. Collineau regards this malady as dependent upon a general, and not upon merely a local affection; it is a disease of the whole system, and not of one part of it. In evidence of this, he points to the well-known fact, that the throat, pharynx, œsophagus, nasal passages, Eustachian tube, larynx, and bronchi, are all either simultaneously or successively attacked, and coated with the morbid secretion. If, therefore, the disease be systemic, how can we expect to oppose it by local applications or by tracheotomy? The alleged success of this operation he considers to be quite illusory; for some cases have terminated favourably of themselves, where little or nothing had been done by art. The only indication for its performance is when all general means have failed, and the patient is threatened with suffocation—

"Melius, remedium anceps, quam nullum."

M. Velpeau advocates the doctrines and defends the practice of M. Bretonneau, who has had by far the most extensive practice in diptheritis, and who, be it remembered, does not recommend tracheotomy except in the last extremity, when there is great danger of asphyxia. The chief object which he has in view by its performance, is the instillation of the solution of the nitras argenti, in order to promote the detachment of the false membranes, and to excite their expulsion from the air-passages.

### A NEW TALIACOTIAN OR PLASTIC METHOD OF TREATING LARYNGO-PHARYNGEAL FISTULÆ.

M. Velpeau recommends a modification of the plan which has hitherto been usually adopted, to retain the raw surfaces in apposition. The irritation of the twisted suture is apt to induce an erysipelas round the wound, and the constant dribbling of the saliva between its lips retards, or may quite prevent, the process of union. Having detached the tegumentary flap, and adjusted it nicely to the fistula, whose edges have been previously made raw, he passes across from left to right a long needle through the lips of the wound, and through the whole breadth of the "bouton" or fleshy plug, thus securing it steadily in the wound; a stitch or two of the simple suture at the apex and root of the flap, are also in some cases necessary.

M. Velpeau has tried the above method in two cases, with perfect success; and is, therefore, induced to recommend it, in preference to all others, in Taliacotian operations, when it is found difficult to retain the plug and the wound closely together.

### CURIOUS MONSTROSITY.

M. Roux related the particulars of a person, who is truly of no visible sex at all. The individual passes for a woman; but there is neither a penis nor a vagina, uterus nor mammæ. The general aspect is certainly more feminine than male; but this is all that can be said.

This person is at present living in Paris.

---

### LXXIV. ACADEMY OF SCIENCES. *(Continued.)*

### *Séance of 1st April.*

### GERMINATION.

MM. Edwards and Becquerel read memoirs on some of the products of the germination of seeds. Their experiments were carried on quite apart from, and unknown to, each other, and we may, therefore, be less scrupulous in admitting the correctness of their results. Both ascertained that acetic acid is generated during germination, and that this evolution goes on for some time after the escape of the radicle and plumule, and as long as the cotyledons continue to exercise any function.

### *Séance of 15th.*

### MORAL STATISTICS OF FRANCE.

M. Guerry, an advocate, has recently published an essay on this subject. Among other topics treated of is that of suicide. It appears that the number of felones de se, from the year 1827 to 1830, committed in France amounts to 6,900, or rather that 6,900 have been registered. The actual number is, therefore, probably much greater, and exceeds, in the proportion of three to one, the number of murders and assassinations of the lives of others.

The frequency of the crime of self-destruction appears to be very different in different divisions of the kingdom; for out of a hundred cases, 51 were committed in the region of the North; 11 in the South; 16 in the East; 13 in the West; and 9 in the central division. In the department of the Seine alone, one-sixth of the whole number takes place, being one in every 3,600 inhabitants; but it is proper to state at the same time, that the greater number of suicides is among the strangers and not the permanent residents in the metropolis; thus, in a hundred cases recorded as having occurred in the region of the North: 21 have been among visitants from the East division; 16 from the South; 7 from the West; 5 from the centre; and therefore only 50, or one-half of the whole number among the permanent resident inhabitants. Still it is a very instructive feature of the researches of M. Guerry, that from whatever point in France we start, we shall find that the proportional frequency of the crime increases, as we approach to Paris. The same remark is applicable to Marseilles, as we may consider that town as the capital of the South. The very reverse holds true of murders and assassinations, which are therefore, proportionally to the number of inhabitants, more numerous, where suicides are the least so.

### Circulation of the Blood.

Dr. Tanchose, in a letter which was read at the Academy, threw out some very ingenious conjectures on the probable agency of a well known law of hydraulics, as a co-operating power along with the contractions of the heart, and perhaps also of the arteries and veins, in sustaining the beautiful mechanism of the circulation.

He supposes that there is constantly and everywhere, but especially in the capillary vessels, a tendency to the formation of a vacuum, induced by the ceaseless withdrawal of some of the constituents of the blood for the purposes of secretion, &c. &c. and that, as a matter of course, there is a steady and uniform afflux or suction of the moving fluid, to all parts of the body. We know that the circulation and the secretions are much accelerated by the rarifaction of the air; the effects of an ordinary cupping-glass are seen every day; and it is well established, that upon any rapid ascent into a thinner atmosphere, hæmorrhages from different parts, especially from the lungs, are by no means unfrequent. Excessive heat produces nearly the same effects; and an opposite state, namely, of cold, and of augmented gravity of the superincumbent air, always retards, and may even quite arrest the circulation and the different secretions. Again, if any part is more than ordinarily excited, a greater quantity of blood is drawn to that part; but it is not the heart, which in this instance, first begins to beat more strongly; the local afflux has already taken place, and the increased action of the heart is consecutive; if the excitement now cease suddenly, the heart still continues for some time after to beat more forcibly and quickly, and regains its tranquil state only gradually, and in proportion as the blood flows back from the part, whither it had been attracted. These facts may be well observed in the effects upon the circulation in the lower extremities, during and after walking; the action of the muscles causes a greater flow of blood, and no doubt a larger expenditure of it.

They are also illustrated by the phenomena of a topical inflammation or phlegmon; the blood-vessels in and around the part are beating strongly and frequently; and the heart becomes affected only when the irritation becomes general.

If the preceding views be correct we cannot fail to be struck with the analogy which will thus exist between the circulation of the blood in animals, and of the sap in vegetables.

M. Isidore Geoffroy St. Hilaire was elected a Member of the Institute in the Section of Anatomy and Zoology. The death of Latreille had occasioned the vacancy.

### Organs of Circulation in the Crocodile.

M. Geoffroy St. Hilaire read a report on the very admirable "tableau," entitled "circulation of the blood in the human fœtus compared with the circulation in the four classes of vertebrated animals," which was recently presented by M. Martin Saint Ange to the Academy of Sciences. At present we shall confine ourselves to one topic only, that of the organs of circulation in the crocodile. Before the researches of M. St. Ange, it was believed that the heart of this gigantic lizard was provided with but one ventricle, divided indeed into three compartments, having numerous orifices in their partitions, and receiving the blood from the right and left auricles, as in the tortoise, where also valves are placed to prevent its return; and that from these different compartments, three great arteries issued, viz. the right and left aorta, and the pulmonary artery.

Now, all this description is ascertained to be erroneous, for the heart of the crocodile most closely resembles that of a mammal; it has two auricles, and two ventricles, and between these cavities there are distinct and perfect septa. So far the resemblance is complete; but upon examining the organs still further, we find that a large artery springs from the right ventricle, at the side of the pulmonary trunk, and communicates by a very short anastomosis, with a branch proceeding from the left ventricle; thus a mixture of the blood from the two sides is effected, but not until the ascending trunks which supply the head are given off; pure arterial blood is therefore sent to the brain, organs of sense, &c. and mixed blood to all the other organs of the body. This discovery of M. St. Ange is most interesting in various points of view, but in none more so than as it most beautifully and unexpectedly illustrates the science of "teratology," or of monstrosities. The eminent English anatomist, Cowper, has recorded the following observations. In the bodies of two young children he found that the aorta, after having formed its arch, and given off the subclavians and carotids, was reduced to an exceedingly small size, nay even partially obliterated, until it was joined by a large branch from the pulmonary trunk; this monstral, or abnormal condition in man, is therefore the normal condition in the crocodile. Before M. St. Ange's discovery, the preceding cases of Cowper were the only examples of anomalies in the vascular system of man which had not their repetition or type in the regular or healthy state of some of the lower animals. That principle, or axiom of philosophical anatomy, that all the "vitia" of the conformation and of the arrangement of the circulatory apparatus in man and in the higher animals are analogous to the normal structure of beings placed lower in the Zoological series, is thus surprisingly confirmed. To compensate for the deficiency of arterialised blood in those parts, which are situated posterior to the heart, or nearer to the tail of the animal, there is a beautiful mechanism, the discovery of which is due to M.M. St. Ange and the younger St. Hilaire. They found a set of tubes, which, opening outwardly close to the recto-sexual passages, penetrate directly into the cavity of the peritoneum; by means of these, air or aerated water may enter and come into contact with certain congeries of blood-vessels in the abdomen; thereby oxygenating their contents, in the same manner as the blood in the bronchial vessels of fishes is arterialised. A subsidiary or local respiration is thus established; and that too, be it remembered, in a part of the body where very important functions, as those of generation, &c. have their seat. These trachea or spiracula have received from the discoverers the name of "peritoneal canals." They have been found also in the testudinata; and are either "aquiferous" or "aeriferous," according to the medium in which the animals live. Analogous, although considerably modified, passages exist in some of the higher animals, as for example, in the monotremata and marsupiata.

The distinguished reporter anticipates from the discovery some highly important and curious results in the field of philosophical zoology.

